# Supplementary material for: Rab27a co-ordinates actin-dependent transport by controlling organelle-associated motors and track assembly proteins
Source: Nat Commun. 2020 Jul 13;11:3495. doi: 10.1038/s41467-020-17212-6 (PMC7359353; doi:10.1038/s41467-020-17212-6)
Supplement: Supplementary file 1 — Supplementary Information [file 41467_2020_17212_MOESM1_ESM.pdf]

**Movie S1.** Image sequence showing output of the 'attached' Cytosim simulation of melanosome transport in melanocytes see main text and Figure 9 for details. Companion to Figure S11A. Acquisition frame rate 0.5 $sec^{-1}$. Playback frame rate 5 $sec^{-1}$.

**Movie S2.** High magnification detail from movie 1 showing the melanosome and actin filament movements in a small area of a model cell (melanosomes are shown individually coloured in order to aid tracking of individuals over time). This region corresponds to the red boxed region shown in the overview images in Figure S11B. Companion to Figure S11B. Acquisition frame rate 0.5 $sec^{-1}$. Playback frame rate 5 $sec^{-1}$.

**Movie S3.** Image sequence showing output of the 'unattached' Cytosim simulation of melanosome transport in melanocytes see main text and Figure 9 for details. Companion to Figure S11A. Acquisition frame rate 0.5 $sec^{-1}$. Playback frame rate 5 $sec^{-1}$.

**Tables**

**Table S1. The complete list of genes targeted by siRNA knockdown in melanocytes in this study.**

| Gene Symbol | Accession # (NM_) | Gene Symbol | Accession # (NM_) | Gene Symbol | Accession # (NM_) |
|---|---|---|---|---|---|
| Actg1 | NM_009609.2 | Rac1 | NM_009007.2 | inf2 | NM_198411 |
| Iqgap1 | NM_027711.1 | Rac2 | NM_009008.3 | arpc1a | NM_019767 |
| Pfn1 | NM_011072.4 | Rac3 | NM_133223.4 | WASp | NM_009515 |
| Msn | NM_010833.2 | RhoBTB1 | NM_001252638.1 | N-WASp | NM_028459 |
| Myh9 | NM_022410.2 | RhoBTB2 | NM_153514.5 | wave1 | NM_031877 |
| Rhoc | NM_007484.2 | RhoBTB3 | NM_028493.2 | wave-2 | NM_153423 |
| Sep-02 | NM_001159719.1 | RhoH | NM_001081105.1 | wave-3 | NM_145155 |
| Mtpn | NM_008098.4 | Rnd1 | NM_172612.3 | wash | NM_001037 |
| Tln1 | NM_011602.5 | Rnd2 | NM_009708.1 | whamm | NM_00100418 |
| Capzb | NM_001037761.2 | RhoD | NM_007485.4 | jmy | NM_021310 |
| Cotl1 | NM_028071.3 | IQgap2 | NM_027711.1 | cortactin | NM_007803 |
| Coro1c | NM_011779.3 | Actr2 | NM_146243.2 | HCLS1 | NM_008225 |
| Myl6b | NM_172259.1 | Arp3 | NM_023735.2 | COBL | NM_172496 |
| Tmod3 | NM_016963.2 | Arpc5 | NM_026369.2 | lmod1 | NM_053106 |
| Actr3 | NM_023735.2 | Arpc4 | NM_026552.3 | lmod2 | NM_053098 |
| Zyx | NM_011777.2 | Arpc2 | NM_029711.1 | lmod3 | NM_001081157 |
| Dstn | NM_019771.2 | Arpc3 | NM_019824.3 | ctnna2 | NM_145732 |
| Epb4.1l3 | NM_013813.1 | Tagln2 | NM_178598.2 | ctnna1 | NM_009818 |
| Epb4.1l2 | NM_001199265.1 | Flna | NM_010227.2 | ctnna3 | NM_001164376 |

| | | | | | |
|---|---|---|---|---|---|
| Ppp1cc | NM_013636.3 | Dsp | NM_023842.2 | ctnnb1 | NM_007614 |
| Rap2b | NM_029519.3 | Ptma | NM_008972.2 | cdh1 | NM_009864 |
| Cdc42 | NM_001243769.1 | Tpm1 | NM_001164256.1 | cdh2 | NM_007664.4 |
| MYOVA | NM_010864.2 | Cfl1 | NM_007687.5 | cdh3 | NM_001037809 |
| Vasp | NM_009499.2 | Actb | NM_007393.3 | Lrrc58 | NM_177093 |
| Enah | NM_010135.2 | Diap1 | NM_007858.2 | acta1 | NM_009606 |
| Diap2 | NM_172493.2 | Diap3 | NM_019670.1 | actg2 | NM_009610 |
| FMNL3 | NM_011711.1 | FMNL1 | NM_019679.2 | actc1 | NM_009608 |
| DAAM1 | NM_026102.2 | FMNL2 | NM_172409.2 | acta2 | NM_007392 |
| DAAM2 | NM_001008231.2 | FHOD1 | NM_177699.4 | actbl2 | NM_175497 |
| FMN1 | NM_010230.2 | FHOD3 | NM_175276.3 | rab27a | NM_023635 |
| FMN2 | NM_019445.2 | RhoB | NM_007483.2 | jup | NM_010593 |
| RhoA | NM_016802.4 | ROCK2 | NM_009072.2 | ctnnd | NM_001085448 |
| Rif | NM_175092.3 | RhoJ | NM_023275.2 | vcl | NM_009502 |
| ROCK1 | NM_009071.2 | RhoG | NM_019566.3 | vim | NM_011701 |
| Spire1 | NM_194355.2 | Rnd3 | NM_028810.2 | Nckipsd | NM_030729 |
| Spire2 | NM_172287.2 | Arpc1b | NM_023142.2 | gsl | NM_146120 |
| RhoQ | NM_145491.2 | Lmo2 | NM_001142336.1 | coro6 | NM_139128 |
| RhoU | NM_133955.4 | grid2ip | NM_133355 | coro1a | NM_009898 |
| RhoV | NM_145530.2 | fhdc1 | NM_001033301 | coro1b | NM_011778 |

| **Gene Symbol** | **Accession # (NM_)** |
|---|---|
| coro2a | NM_178893 |
| wipf1 | NM_153138 |
| nckbeta | NM_010879 |
| pak1 | NM_011035 |
| Tmod1 | NM_021883 |
| tmod2 | NM_001038710 |
| tmod4 | NM_016712 |
| tpm2 | NM_009416 |
| tpm3 | NM_022314 |
| tpm4 | NM_001001491 |

**Table S2: Rab27a unique peptides identified using mass spectrometry at ≥95% confidence level in anti-Rab27 immunoprecipitates.**

| **Confidence %** | **Sequence** | **Modifications** | **Protein Modifications** | **Cleavages** | **Obs MW** | **z** |
|---|---|---|---|---|---|---|
| 99 | DAMGFLLLFDLT NEQSFLNVR | Carbamido methyl @N-term | | | 2499.2451 | 3 |
| 99 | FITTVGIDFR | | | | 1167.6279 | 2 |

| | | | | | | |
|---|---|---|---|---|---|---|
| 99 | FLALGDSGVGK | Carbamido methyl @N-term | | | 1119.5885 | 2 |
| 99 | FLALGDSGVGK | | | | 1062.5676 | 2 |
| 99 | IHLQLWDTAGQER | | | | 1565.7892 | 3 |
| 99 | LQLWDTAGQER | Carbamido methyl @N-term | | cleaved H-L@N-term | 1372.6766 | 2 |
| 99 | LQLWDTAGQER | | | cleaved H-L@N-term | 1315.6528 | 2 |
| 99 | SDGDYDYLIK | Acetyl@N-term | Acetyl@2 | cleaved M-S@N-term | 1229.5472 | 2 |
| 99 | SLTTAFFR | | | | 941.4979 | 2 |
| 99 | SNGHTSADQLSEEK | Ammonia-loss(N)@2 | | | 1484.6572 | 2 |
| 99 | TSVLYQYTDGK | | | | 1273.6177 | 2 |
| 99 | SWIPEGVVR | | | | 1041.5584 | 2 |
| 99 | ANGPDGAVGR | | | | 912.4656 | 2 |

**Table S3: SPIRE1 unique peptides (human) identified using mass spectrometry at ≥95% confidence level in anti-Rab27 immunoprecipitates.**

| Confidence (%) | Sequence | Modifications | Cleavages | Obs MW (Da) | z |
|---|---|---|---|---|---|
| 99 | ALFAETMELHTFLTK | | | 1750.889 | 3 |
| 99 | APTLAELDSSESEEETLHK | | | 2084.971 | 3 |
| 99 | DALSLEEILR | | | 1157.628 | 2 |
| 99 | DGAVTLAPAADDAGEPPPVAGK | | | 2018.018 | 2 |
| 99 | ENGLSTSQQVPAQR | | | 1513.742 | 2 |
| 99 | ENGLSTSQQVPAQR | Deamidated(N)@2 | | 1514.732 | 2 |
| 99 | EPGAAGGAAGGSR | | | 1056.495 | 2 |
| 99 | FLPISSTPQPER | | | 1370.713 | 2 |
| 99 | FWVQVMR | | | 964.5029 | 2 |
| 99 | LCAAHLPTESDAPNHYQAVCR | Carbamido methyl(C)@2 Carbamido methyl(C)@20 | | 2409.098 | 4 |
| 99 | LPSKPYSTLPIFSLGPSALQR | | | 2271.262 | 3 |
| 99 | NLVESSMVNGGLTSQTK | Deamidated(N)@9 | | 1764.897 | 2 |
| 99 | SAHEIILDFIR | | | 1312.713 | 2 |
| 99 | SDESSTDLEELK | | | 1351.599 | 2 |

| | | | | | |
|---|---|---|---|---|---|
| 99 | SMDKSDEELQFPK | | missed K-S@4 | 1552.695 | 3 |
| 99 | STLPIFSLGPSALQR | | cleaved Y-S@N-term | 1585.882 | 2 |
| 99 | STSSSSVSPSFPEEPVLEAVSTR | | | 2379.126 | 3 |
| 99 | TEAVGGEGPR | | | 971.4687 | 2 |

**Table S4. The sequences of siRNA oligonucleotides used in this study.**

| **siRNA name** | **Sense oligonucleotide sequence 5'-3'** |
|---|---|
| SPIRE1#1 | GGAAACACCUACGAACGUG |
| SPIRE1#2 | GAAAUUAGACGGAGCAGAC |
| SPIRE1#3 | GGACGACAUUCGGUGCAAA |
| SPIRE1#4 | GCAUACAUUUCUGACCAAA |
| SPIRE2#1 | GGACGCACAUGAACUUAUC |
| SPIRE2#2 | GAGCGUGGAUGUCCUCAAU |
| SPIRE2#3 | CAAGAAGUAUGGACACAUC |
| SPIRE2#4 | CAAAGAACACUGCACGAGA |
| Formin-1#1 | GGACAAACCUGAACAAUUU |
| Formin-1#2 | GAACGUGCCCAGUGCAUAA |
| Formin-1#3 | GCAGACGGAUAUAGUUUAG |
| Formin-1#4 | GGUGAGCACGCAUUAGUAA |
| Rab27a | UUGAGAAUCCAGAUUUCAA |
| NT | UAAGGCUAUGAAGAGAUAC |

**Table S5. The sequences of taqman primers and probes used in this study.**

| **Target** | **Sense strand primer 5'-3'** | **Reverse strand primer 5'-3'** | **Probe 5'-3'** |
|---|---|---|---|

| | | | |
|---|---|---|---|
| GAPDH | GTGTCCGTCGTGGATCTGA | CCTGCTTCACCACCTTCTTGA | CCGCCTGGAGAAACCTGCC AAGTATG |
| SPIRE1 | GGCTATGTATTATCTGTTTG AGAGAGCTT | CCAGGACCTGACGGATGTG | AACCCTCAAAGGAGGAATT CTGCTACCCA |
| SPIRE2 | TCCGTGCCAGGAACTATAA GCT | GATAAGTTCATGTGCGTCCTT CTTC | CAAGGTCATGGTCGATG |

**Table S6. Primers and control templates used in qRT-PCR experiments.**

| **Target** | **Sense strand primer 5'-3'** | **Reverse strand primer 5'-3'** | **Template** |
|---|---|---|---|
| SPIRE1 | AGGATGAAGGGTACGAGGCT | CTGATAATGATTGGGTGCCTCC | pAcGFP-C1-mm-SPIRE1 (Full-length murine SPIRE1 (XM_006526207.3 ), as a C-terminus fusion to GFP) |
| SPIRE2 | ACACTGCACGAGAAGATCCT | ACTTGATGTCCCCAGAGCAG | pAcGFP-C1-mm-SPIRE2 (Full-length murine SPIRE2 (AJ459115), as a C-terminus fusion to GFP) |
| FMN1 | AGCTGGTGTGCAAGGAGTCC | GCTGGGGGTGACCTCCTTCT | pcDNA3-mm-FMN1-FH2 (FH2 domain of murine FMN1 XM_011239295.2) |
| FMN2 | TTCACAGGGAGAACACTGCT | ACTGCCCTTCGCTGTAATCC | pEGFP-C1-mm-FMN2 Full-length murine FMN2 (NM_019445.2), as a C-terminus fusion to EGFP) |
| Mlph | TTGAACAAGCGAATGTCAGCTG TGG | GAGTGTCTCCTCCTCTGTGTCAGC A | pAcGFP1-C1-mm-Mlph (aa 2 - 466 of murine Mlph (NM_053015.3), |

| | | | |
|---|---|---|---|
| | | | as a C-terminus fusion to GFP) |

**Table S7. Primary antibodies used in this study.**

| Antibody | Supplier | Catalogue code | Dilution/ Concentration (application) | Figure |
|---|---|---|---|---|
| Rabbit anti-FMN1 | Markus Dettenhofer (CEITEC - Central European Institute of Technology, Brno, Czech Republic.) | [1] | 1:1000 (immunoblot) | 1d |
| Goat anti-GAPDH | Sicgen | Ab0049-200 | 1:5000 (immunoblot) | 1d, S4c |
| Goat anti-GFP | Sicgen | AB0020-200 | 1:1000 (immunoblot) | S4 |
| Rabbit anti-GFP | TakaraBio/Clontech | 632376 | 1μg/ml (immunoblot) | 6e S5c S6 |
| Mouse monoclonal anti-c-Myc (9E10) | Santa Cruz Biotechnology | sc40 | 0.4μg/ml (immunoblot) | 6b, 6c |
| Mouse monoclonal anti-TRP1 (Ta99) | Santa Cruz Biotechnology | sc-58438 | 1:1000 (immuno-fluorescence) | S8b |
| Mouse monoclonal anti-GFP IgG1κ (clones 7.1 and 13.1) | Sigma-Aldrich | 11814460001 | 1:300 (immuno-fluorescence), 1:1000 (immunoblot) | 1f, 1g, 4b, 5b, 7, 8b, S1g, S3b, S5e, S9a, S10b, S10d |
| Mouse-anti-human HSP90 | Santa Cruz, Biotechnology. | sc-69703 | 1:1,000 (immunoblot) | S10d |
| Rabbit-anti-human ARL1 | Proteintech. | 16012-1-AP | 1:2,000 (immunoblot) | S10d |

**Table S8. Seconday antiobies used in this study.**

| Antibody | Conjugate | Supplier | Catalogue code | Dilution/ Concentration (application) | Figure |
|---|---|---|---|---|---|
| Donkey anti-Rabbit IgG | IRDye® 800CW | LI-COR Biosciences, Cambridge, UK. | 926-32213 | 1:10,000 | 1d |
| Donkey anti-Goat IgG | IRDye® 800CW | LI-COR Biosciences, Cambridge, UK. | 926-32214 | 1:10,000 | 1d, S4 |

| | | | | | |
|---|---|---|---|---|---|
| donkey anti-rabbit IgG | Horseradish peroxidase | GE Healthcare Life Sciences,. | 10794347 NA934 | 1:5000 | 6e S5c S6 |
| sheep anti-mouse IgG | Horseradish peroxidase | GE Healthcare Life Sciences, | 10094724 NXA931 | 1:5000 | 6b, 6c |
| Goat anti-mouse | Alexa568 | Invitrogen | A-11011 | 1:500 | 1f, 1g, 4b, 5b, 7, 8b, S1g, S3b, S5e, S9a, S10b |
| Donkey anti-mouse | Cy5 | Dianova | 715-175-151 | 3.25μg/ml | S8b |
| Goat anti-mouse | Horseradish peroxidase | Advansta | R-05071-500 | 1:10,000 | S10d |
| Goat anti-rabbit | Horseradish peroxidase | Advansta | R-05072-500 | 1:10,000 | S10d |

**Table S9. Prokaryotic and eukaryotic expression vectors used here**. Unless otherwise stated vectors allow expression of protein in mammalian cells and were generated in this study. pENTR vectors were used to generate adenoviruses [2].

| Vector | Expressed protein |
|---|---|
| pENTR-EGFP | EGFP alone [2]. |
| pENTR-EGFP-C2-mm-FMN1, FH1-FH2-FSI, N-term, ΔFSI, FH2-FSI | Murine full-length FMN1 and aa849-1430, aa1-859, aa1-1394, aa985-1430 fragments as a fusion to the C-terminus of EGFP. Generated by PCR and sub-cloning from pEGFPC2-FMNIso1b (Addgene #19320) (XP_011237597) [3]. |
| pENTR-EGFPC1-Myc-hs-SPIRE1 | Human SPIRE1 (KIAA1135) as a fusion to the C-terminus of EGFP-Myc-epitope. |
| pENTR-EGFPC1-Myc-hs-SPIRE2 | Human SPIRE2 (AJ422077) as a fusion to the C-terminus of EGFP-Myc-epitope. |

| | |
|---|---|
| pGEX-4T1-rn-Rab27a (-WT, -Q78L, -T23N) | Wild-type, active and inactive mutant forms of rat Rab27a (P23640) in *E.coli* as fusions to the C-terminus of GST. |
| pcDNA3-Myc-hs-SPIRE1, KWM and MSFH | Full-length human SPIRE1 (KIAA1135), aa2-524 and aa388-742 fragments as a fusion to the C-terminus of the Myc-epitope. |
| pcDNA3-Myc-hs-SPIRE2 and MSFH | Full-length human SPIRE2 (AJ422077) and aa361-714 fragment as a fusion to the C-terminus of the Myc-epitope. |
| pAcGFP1-C1-hs-SPIRE1-MSFH, SFH, SF, FYVE and SB | Full-length human SPIRE1 (KIAA1135), aa388-742, aa525-742, aa525-697, aa573-697 and aa525-572 fragments as a fusion to the C-terminus of AcGFP1. |
| pCMVMYC-mCherry | Myc-mCherry fusion protein [4]. |
| pENTR-V5-vYn Myc-hs-SPIRE1 and hs-SPIRE2 | Full-length human SPIRE1 and SPIRE2 as a fusion to the C-terminus of the V5 epitope tag and an N-terminus fragment (ACE74713) of Venus YFP. |
| pENTR-V5-vYc- rn-Rab27a, T23N, Q78L, N133I | Rat Rab27a as a fusion to the C-terminus of the V5 epitope tag and an C-terminus fragment (AM779184.1) of Venus YFP. |
| pAcGFP1-C1-mm-Mlph RBD | Rab27a binding domain of murine Mlph (aa 1-150; NP_443748.2) as a C-terminus fusion to GFP. |
| pENTR-EGFPC2-mm-MyoVa1-920aa-Sytl2-a RBD | Active S1 (motor-lever) fragment of murine myosin-Va fused at the N-terminus to GFP and |

| | |
|---|---|
| | the C-terminus to the Rab27a binding domain of murine Sytl2-a, described previously [5]. |
| pENTR-EGFPC2-mm-MyoVa1-920aa-SPIRE1/2 | As above but with Sytl2-a replaced with either murine SPIRE1 or SPIRE2. Murine SPIRE coding sequence was PCR amplified from EST clones IRAVp968A10102D (Q52KF3.1) and IRAVp968C09162D (NP_758491.1) obtained from Source Bioscience, Nottingham, UK. |
| pENTR-EGFPC1-hs-SPIRE2 MSFH, KW, WMSFH and KMSFH | Human SPIRE2 (AJ422077) protein fragments; aa 361-714, aa1-370, aa230-714, aa2-230 with 361-714. |
| pENTR-EGFPC1 hs-SPIRE2-KW-Rab27a | Human SPIRE2 (AJ422077) aa1-370 fused to the C-terminus of EGFP and the N-terminus of Rab27aSF1F4 [6]. |
| pENTR-EGFPC2-mm-FMN1-FH1-FH2-Rab27a-SF1F4 | Murine FMN1 fragment aa840-1401 fused to the C-terminus of EGFP and the N-terminus of Rab27aSF1F4 [6]. |
| pENTR-EGFPN3-mm-FMN2, pENTR-EGFPC1-mm-FMN2 | Murine FMN2 full length (NP_062318.2) fused to the N- and C-termini of EGFP. |
| pENTR-EGFPC1-mm-FMN2-FH1-FH2-FSI | Murine FMN2 aa854-1587 fused to the C-terminus of EGFP. |
| pEGFPC3-rn-Rab27a | Rat Rab27a as a fusion to the C-terminus of EGFP [7]. |
| pmRuby3-C1-rn-Rab27a | Rat Rab27a as a fusion to the C-terminus of mRuby3. |

| | |
|---|---|
| pEGFP-C2-mm-MyoVa-CC-GTD | Murine myosin Va fragment (aa 1260-1880) as a fusion to the C-terminus of EGFP [8]. |

## Supplementary Figure 1

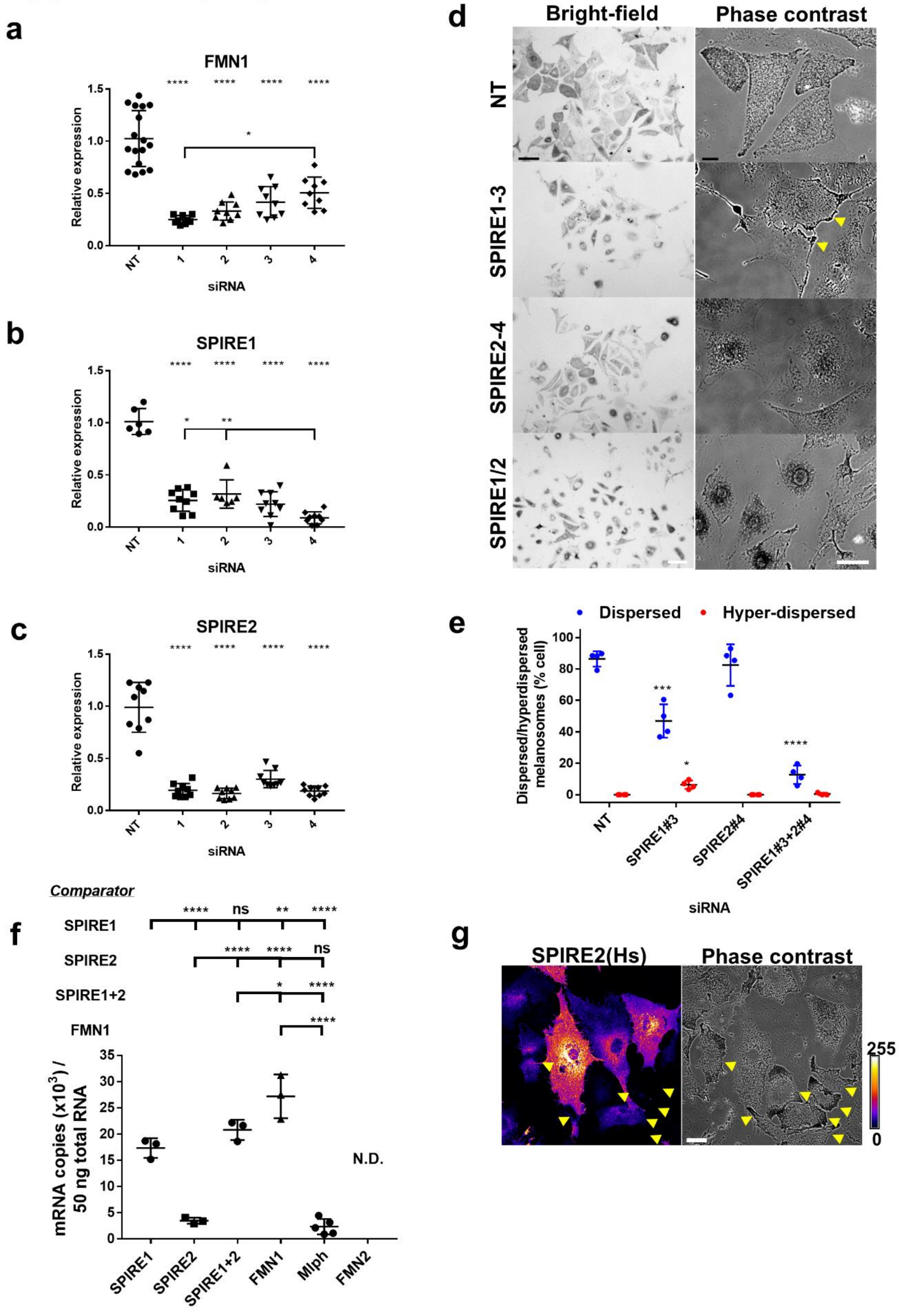

**Figure S1. The effect of siRNA transfection on FMN1 and SPIRE1/2 mRNA and melanosome dispersion. a-c**) Bee swarm plots showing the effect of transfection of melan-a cells with FMN1, SPIRE1 and SPIRE2 specific siRNA oligonucleotides on mRNA levels of the target as measured using Taqman RT-PCR (see Experimental procedures). Each point represents the Taqman assay signal normalised to the mean signal of the NT (non-targeted) control siRNA transfection. **d**) Low and high magnification images showing the distribution of melanosomes in populations of cells transfected using the indicated siRNA directed against SPIRE1 and -2 alone or in combination. Arrows in phase contrast images indicate examples of cells manifesting hyper-dispersed melanosomes. Scale bars = 100 and 20 μm for bright-field and phase contrast images. **e**) A bee swarm plot showing the percentage of siRNA transfected cells in which melanosomes are classed as dispersed or hyper-dispersed. Data are from one experiment and are representative of 3 independent experiments. **f**) A bee swarm plot showing the results of quantitative RT-PCR analysis of the expression of SPIRE1/2, FMN1/2, and MLPH genes in melan-a cells. Data are from 3 independent experiments. N.D. indicates not detected. **g**) Confocal fluorescence and phase contrast micrographs (single *z*-sections) show the distribution of hsSPIRE2 (displayed using 'fire' LUT to highlight cells expressing low levels of SPIRE2) and melanosomes in SPIRE1/2 depleted melan-a cells. Arrows indicate cells with hyper-dispersed melanosome distribution. Scale bar represents 20 μm. **a-c**, **e-f**) ****, ***, ** and * indicate significant difference p =< 0.0001 and p =< 0.001, p =< 0.01 and p =< 0.05 as determined by one-way ANOVA. No other significant differences were seen. Significance indicators at the top of each plot show significance of difference compared with NT control. Horizontal bars indicate the other pairs of data sets that showed significant differences. No other significant differences were observed. Bars indicate the mean and 25$^{th}$ and 75$^{th}$ percentile of data. Source data for a-**c, e** and **f** are provided in the supplementary Source Data file.

## Supplementary Figure 2

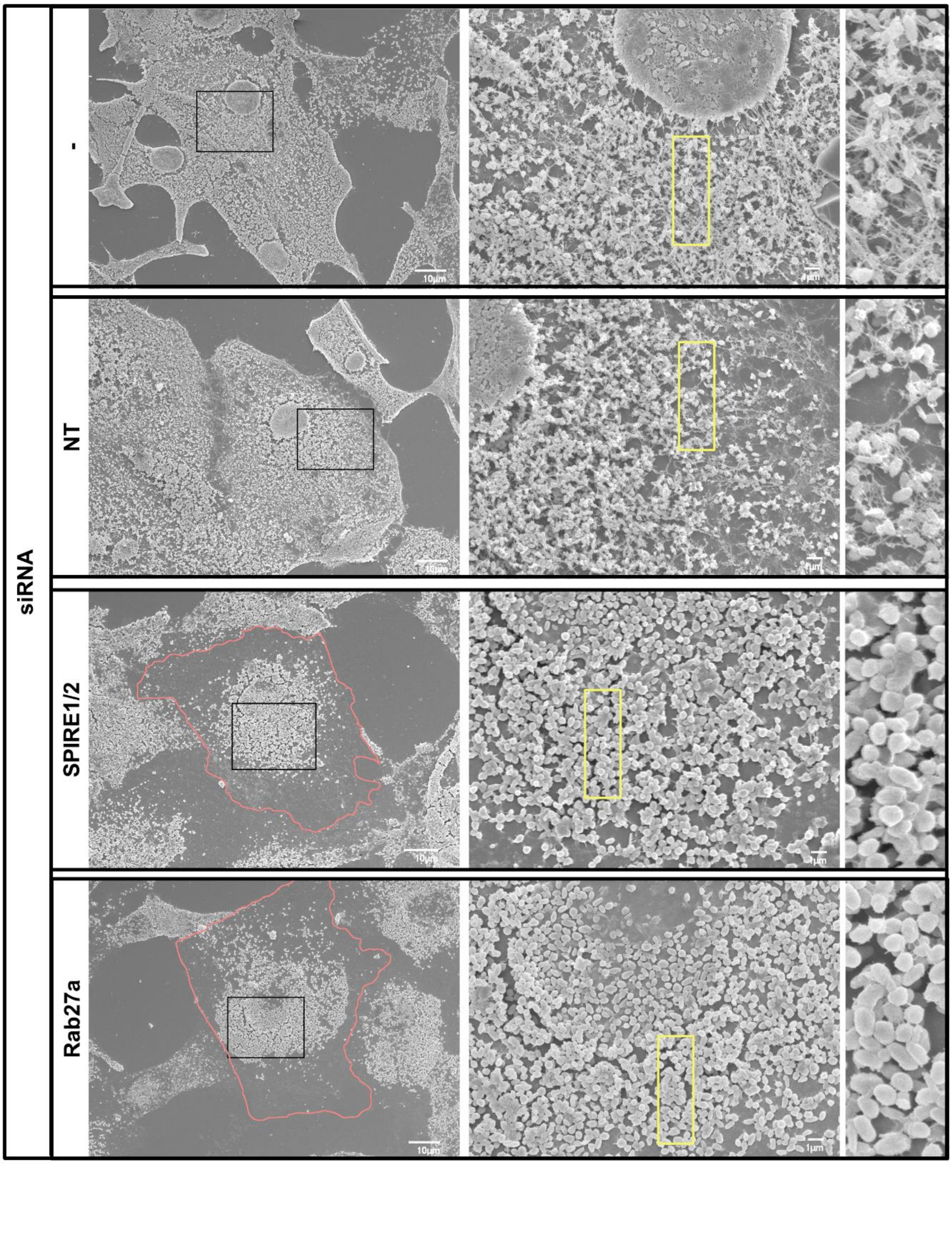

**Figure S2. Scanning electron microscopy reveals a reduction in melanosome associated AFs in SPIRE1/2 and Rab27a depleted melanocytes compared with controls.** Wild-type (melan-a) cells were transfected with the indicated siRNA before fixation and preparation for FESEM (see Experimental procedures). Boxes in low/medium magnification (left/central) images indicate the regions shown in high magnification images to their right. Scale bars = 10 and 1 μm in left and central images.

## Supplementary Figure 3

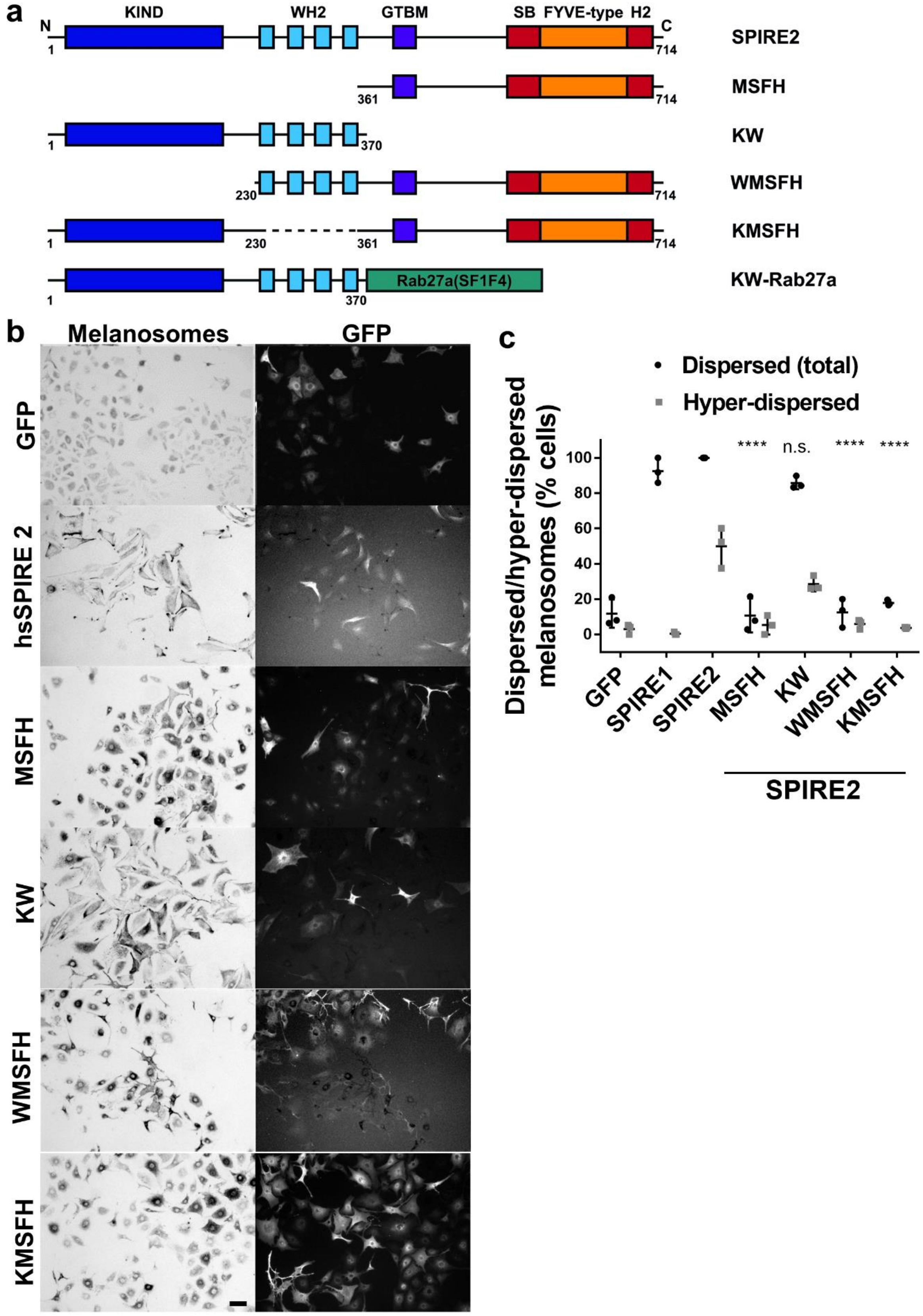


**Figure S3. The FMN interaction (KIND) and AF nucleation (WH2) activities of SPIRE2 are essential for**

**melanosomes dispersion. a**) A schematic representation of the domain structure of human SPIRE2 and the correspondence with truncations and chimeric proteins used in functional studies (**b**-**c**). Numbers indicate amino acid boundaries. *K*, KIND; *W*, WH2; *M*, GTBM, globular tail domain binding motif; *S*, SB, SPIRE-box; *F*, FYVE-type zinc finger; *H*, C-terminal flanking sequences similar to H2 of Mlph-type Rab27 effectors . **b**) melan-a cells were depleted of SPIRE1/2 by siRNA transfection and 72 hours later infected with adenoviruses expressing the indicated proteins. Cells were fixed 24 hours later, processed for immunofluorescence and imaged using bright-field and fluorescence optics to observe melanosome and protein distribution/expression (see Experimental procedures). Scale bars = 50 μm. **c**) Is a bee swarm plot showing the percentage of human SPIRE2 expressing SPIRE1/2 siRNA depleted melan-a cells in which melanosomes are classed as dispersed and/or hyper-dispersed. **** and n.s. indicate significant difference $p = < 0.0001$ and no significant difference as determined by one-way ANOVA in % dispersed-type cells (total) between the test population and the control population of SPIRE2 expressing cells. Results shown are from 3 independent experiments. Bars indicate the mean and 25$^{th}$ and 75$^{th}$ percentile of data. Source data for **c** are provided in the supplementary Source Data file.

## Supplementary Figure 4

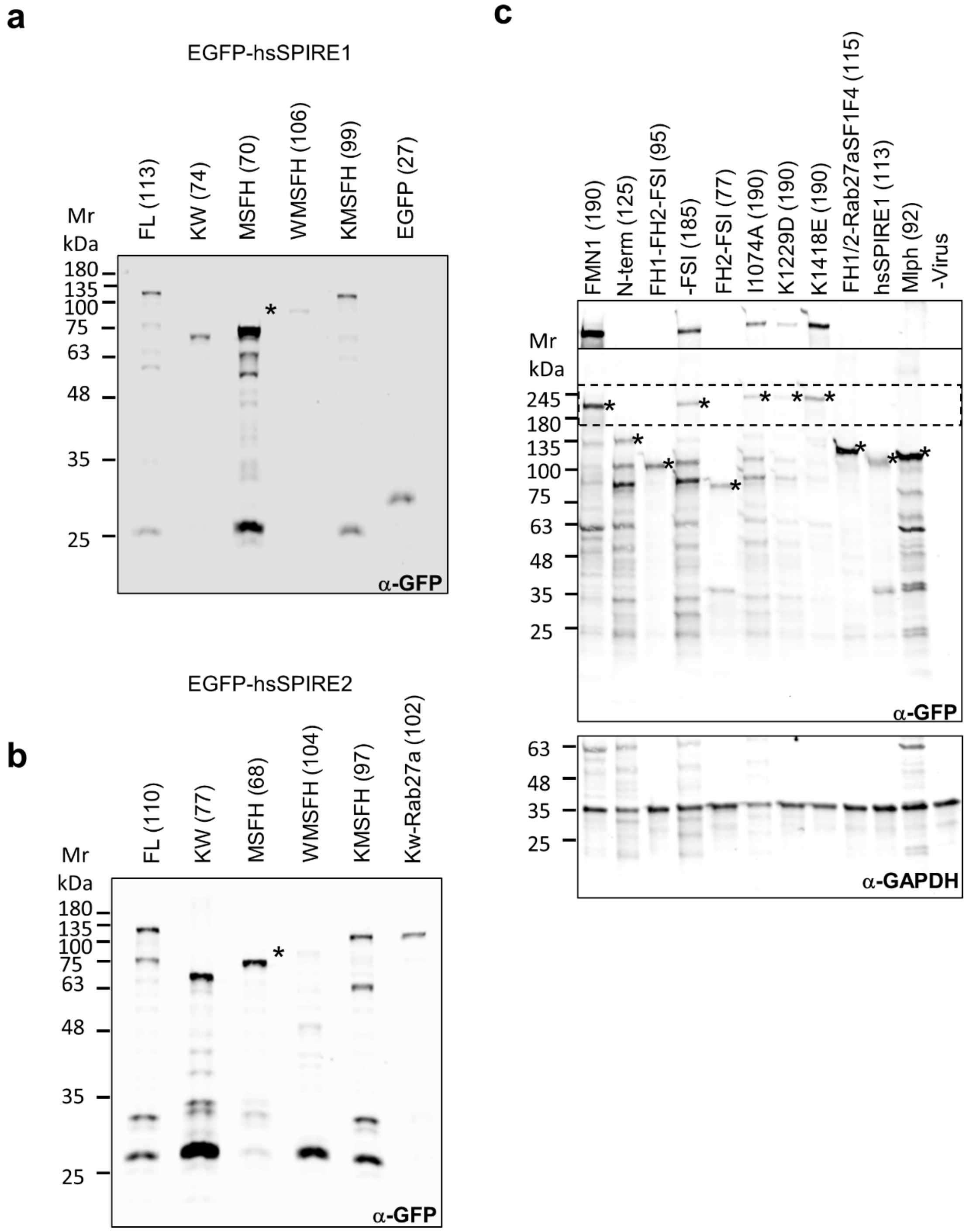

**Figure S4. Confirmation of the expression of SPIRE1/2, FMN1 and mutant proteins in melanocytes**. melan-a (**a**-**b**) and melan-f (**c**) melanocytes were infected with viruses expressing the indicated proteins as GFP fusions. Cells were harvested 48 hours later, whole cell lysates were generated and GFP fusion proteins detected by immunoblotting (see Experimental Procedures).The dotted box in **c** indicates the region shown above using a longer exposure time to improve the visibility of faint bands. Calculated molecular masses, based on amino acid sequences, are indicated in brackets adjacent to the protein name. In **c** lysates were also blotted with anti-calnexin as a loading control. Asterisks indicate the bands that are likely to correspond to each full-length GFP fusion protein based on their primary structure (**a** and **b** to the left of the band and **c** to the right on the band). Source data for **c** are provided in the supplementary Source Data file.

## Supplementary Figure 5

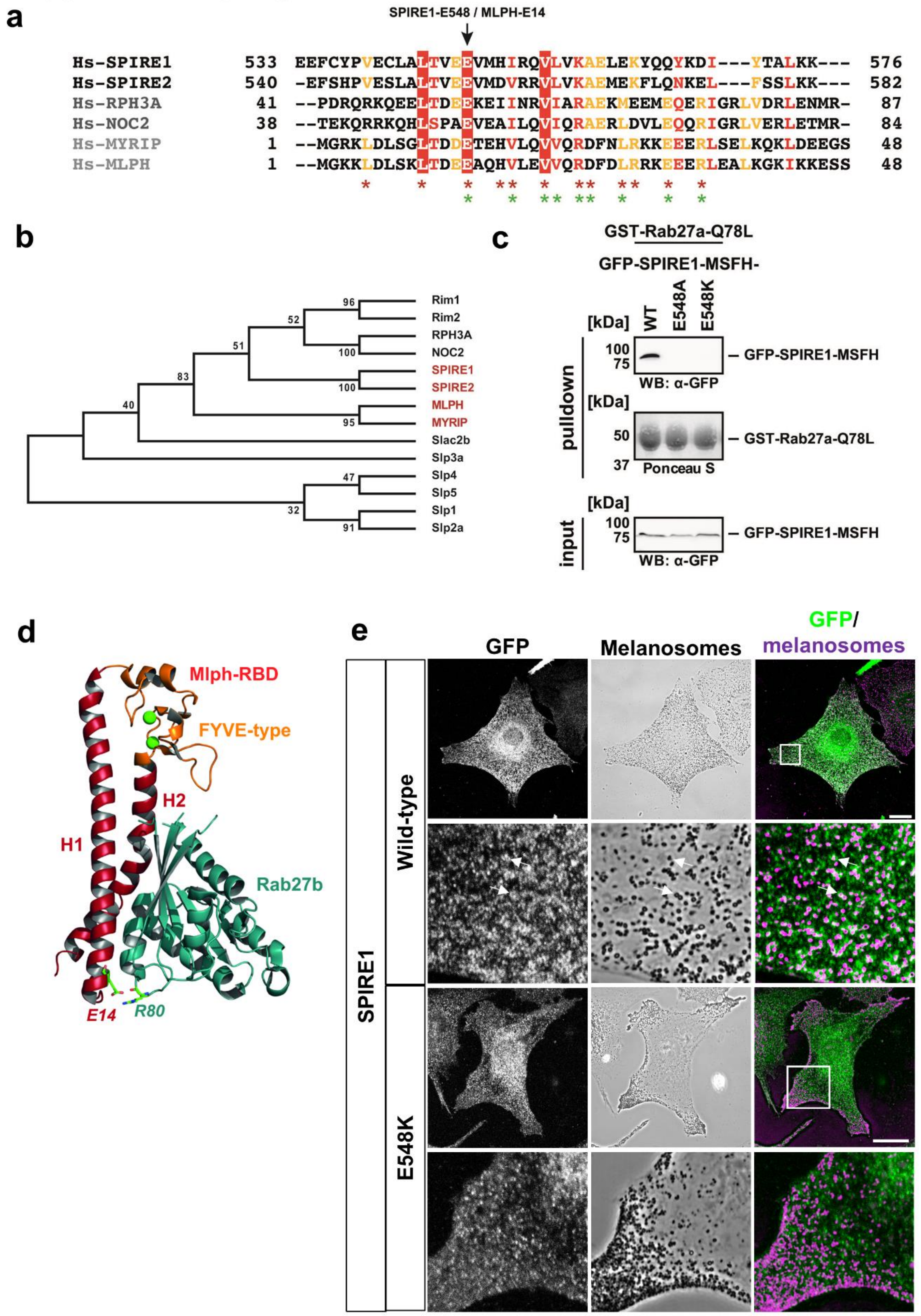

**Figure S5. Comparison of the membrane binding C-terminus of SPIRE1/2 with the Rab27 binding domains of Slp/Slac effectors and generation of the Rab27 interaction deficient SPIRE1$^{E548K}$ mutant**. **a**) A multiple protein sequence alignment of the H1/SB portions of human (Hs) SPIRE and the indicated Slp/Slac class Rab27/3 effectors. Asterisks below the alignment indicate contact residues of Mlph (red) and rabphilin3a (green) in complex with Rab27b and Rab3a [9,10]. Lettering indicates residues that are highly (red) or somewhat (brown) conserved among SPIREs and other effectors. Red shaded letters are fully conserved among Rab27/3 effectors and whose replacement with alanine results in reduced SPIRE1/Rab27a interaction. Numbers on the right indicate amino acids for the respective sequence. **b**) Phylogenetic tree of the Rab27 binding domains of Rab27 effectors Rim1/2, Rabphilin3A, Noc2, Mlph, Myrip, Slac2b/Exophilin, Slp1/2a/3a/4/5 and the SPIRE1 and SPIRE2 proteins (see experimental procedures for accession numbers). The evolutionary history was inferred using the *Maximum Likelihood* method based on the JTT matrix-based model. The bootstrap consensus tree inferred from 500 replicates is taken to represent the evolutionary history of the taxa analysed. Branches corresponding to partitions reproduced in less than 50% bootstrap replicates are collapsed. The percentage of replicate trees in which the associated taxa clustered together in the bootstrap test (500 replicates) are shown next to the branches. **c**) A GST-pulldown assay with purified GTP-locked GST-Rab27a-Q78L and HEK293 cell lysates transiently over-expressing AcGFP1-tagged C-terminal (GFP-SPIRE1-MSFH) human SPIRE1 and mutants. Protein expression levels are shown (input) and Ponceau S staining shows equal amounts of GST-Rab27a-Q78L. N = 2-3 experimental repeats. WB = Western blotting. **d**) Ribbon representation of Rab27B/Mlph-RBD complex structure (PDB ID: 2ZET) showing the position of Mlph E14 and Rab27B R80 and their possible electrostatic interaction [9]. **e**) The distribution of SPIRE1 wild-type and E548K protein and melanosomes in melan-a cells. Cells expressing GFP-SPIRE1 fusion proteins were processes for confocal microscopy (see Experimental Procedures). Images are single confocal z-sections. Scale bars = 20 μm. Boxes in low magnification images (upper trio for each protein) indicate the region shown below in high magnification images.

Arrows indicate association between melanosomes and SPIRE1 protein. Source data for **c** are provided in the supplementary Source Data file.

## Supplementary Figure 6

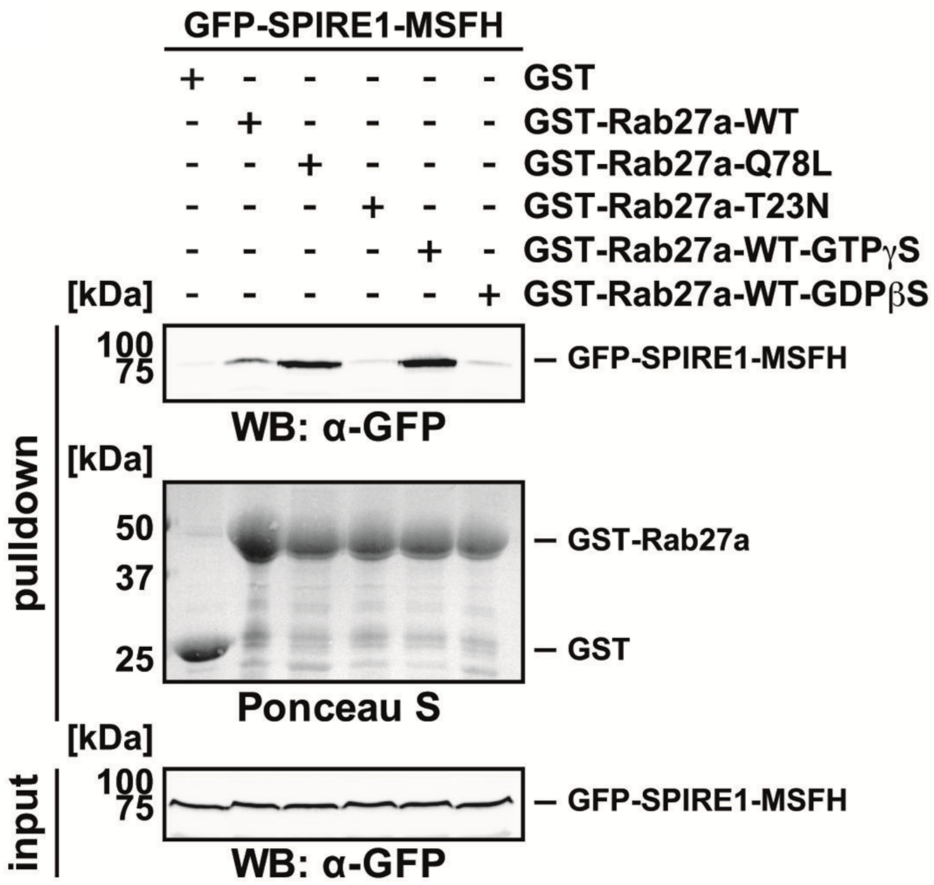

**Figure S6. Nucleotide dependent interaction of Rab27a with the membrane binding C-terminus module of SPIRE1.** GST-pulldown assay with purified GST-Rab27a wild-type (WT), GTP-locked (Q78L and WT-GTP$\gamma$S), GDP-locked (T23N and WT-GDP-$\beta$S), GST alone as control and HEK293 cell lysates transiently over-expressing AcGFP1-tagged C-terminal SPIRE1 (GFP-SPIRE1-MSFH). Respective protein expression levels are shown (input) and Ponceau S staining shows equal amounts of GST-Rab27a proteins. N = 2 experimental repeats. WB indicates Western blotting. Source data are provided in the supplementary Source Data file.

## Supplementary Figure 7

**a**

M**SDGDYDYLIKFLALGDSGVGKTSVLYQYTDGK**FNSK**FITTVG**
**IDFR**EKRVVYR**ANGPDGAVGR**GQR**IHLQLWDTAGQER**FR**SLTT**
**AFFRDAMGFLLLFDLTNEQSFLNVR**NWISQLQMHAYCENPDIV
LCGNKSDLEDQRAVKEEEARELAEKYGIPYFETSAANGTNISH
AIEMLLDLIMKRMERCVDK**SWIPEGVVRSNGHTSADQLSEEK**E
KGLCGC

**b**

MAQAAGPAGGGEPR**TEAVGGEGPREPGAAGGAAGGSRDALSLE**
**EILR**LYNQPINEEQAWAVCYQCCGSLRAAARRQPRHRVRSAA
QIRVWR**DGAVTLAPAADDAGEPPPVAGK**LGYSQCMETEVIESL
GIIIYKALDYGLKENEERELSPPLEQLIDHMANTVEADGSNDE
GYEAAEEGLGDEDEKRKISAIRSYRDVMK**LCAAHLPTESDAPN**
**HYQAVCRALFAETMELHTFLTK**IKSAKENLKKIQEMEK**SDESS**
**TDLEELK**NADWAR**FWVQVMR**DLRNGVKLKKVQERQYNPLPIEY
QLTPYEMLMDDIRCKRYTLRKVMVNGDIPPRLKK**SAHEIILDF**
**IR**SRPPLNPVSARKLKPTPPRPRSLHERILEEIKAERKLRPVS
PEEIRRSRLAMRPLSMSYSFDLSDVTTPESTK**NLVESSMVNGG**
**LTSQTKENGLSTSQQVPAQR**KKLLR**APTLAELDSSESEEETLH**
**KSTSSSSVSPSFPEEPVLEAVSTR**KKPPK**FLPISSTPQPER**RQ
PPQRRHSIEKETPTNVRQFLPPSRQSSRSLEEFCYPVECLALT
VEEVMHIRQVLVKAELEKYQQYK**DIYTALK**KGKLCFCCRTRRF
SFFTWSYTCQFCKRPVCSQCCKKMR**LPSKPYSTLPIFSLGPSA**
**LQR**GESSMRSEKPSTAHHRPLRSIARFSSKSK**SMDKSDEELQF**
**PK**ELMEDWSTMEVCVDCKKFISEIISSSRRSLVLANKRARLKR
KTQSFYMSSPGPSEYCPSERTISEI

**Figure S7. Sequence coverage of the unique peptides identified (≥95% confidence level) via mass spectrometry in Rab27a mouse (a) and SPIRE1 human (b).** The peptide sequence coverage identified for each protein is high-lighted in green. Grey text is the complete protein sequence in each case.

## Supplementary Figure 8

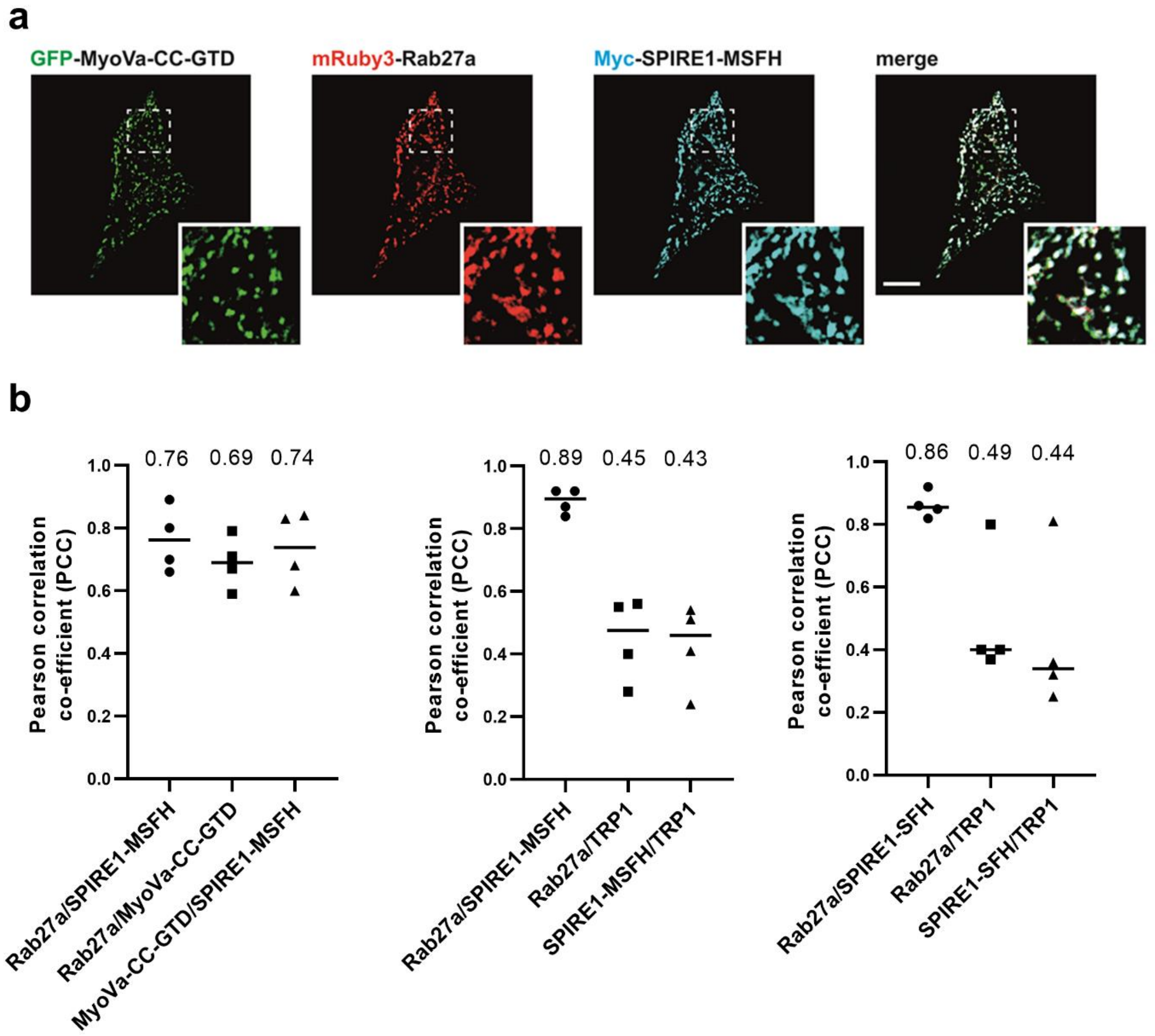


**Figure S8. Colocalisation analysis of Rab27a, SPIRE1, myosin-Va and the melanosome resident**

**protein TRP1 in melanocytes. a**) The localization of transiently co-expressed tagged MyoVa-CC-GTD (eGFP, eGFP-MyoVa-CC-GTD; green), Rab27a (mRuby3, mRuby3-Rab27a; red), and the Myc-epitope tagged (Myc, cyan) C-terminal SPIRE1 protein (SPIRE1-GTBM-SB-FYVE-H2, Myc-SPIRE1-MSFH) was analyzed by fluorescence microscopy. Deconvolved images indicate the localization of the proteins on vesicular structures. Boxes indicate regions shown in high magnification inset images. Scale bar represents 10 µm. 5 cells were recorded for each condition and one representative cell is presented here. **b**) The colocalization of tagged proteins as described in **a**) was quantified by determining the Pearson's correlation coefficient (PCC) as shown in a bar diagram (left panel). The PCC was determined for colocalization of Rab27a and C-terminal SPIRE1 proteins depending on their myosin V binding capacity (SPIRE1-MSFH, middle panel vs. SPIRE1-SFH, right panel) and the extent of localization of both, Rab27a and SPIRE1 proteins, at melanosome membranes (TRP1) was calculated. Each bar represents the mean PCC value for 4 cells analysed, mean values are indicated and error bars represent SEM. Source data for **b** are provided in the supplementary Source Data file.

# Supplementary Figure 9

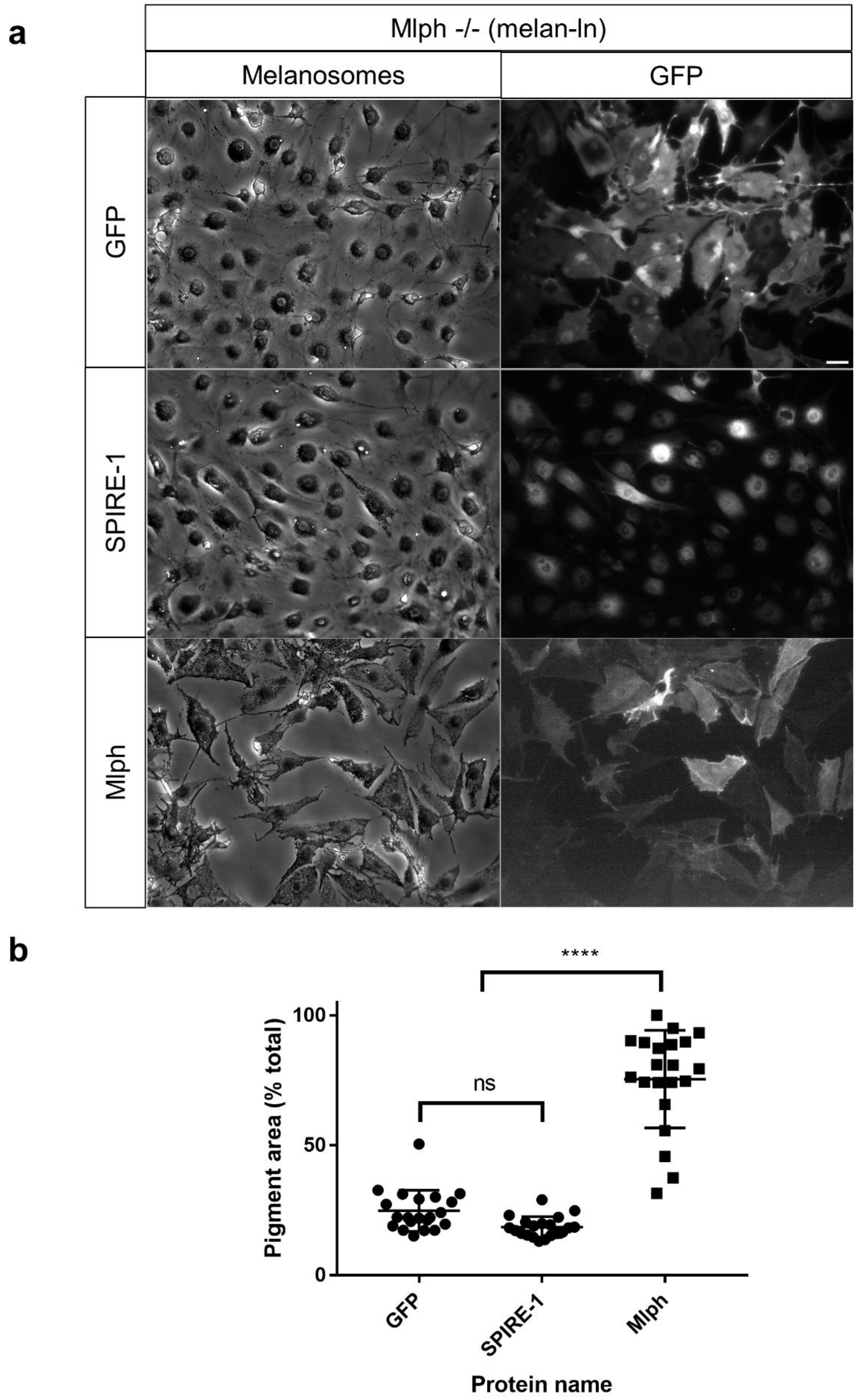


**Figure S9. Expression of SPIRE1 does not restore dispersed melanosome distribution in melan-ln**

**(Mlph -/-) melanocytes.** melan-ln cells were infected with adenoviruses expressing the indicated GFP fusion proteins, fixed 24 hours later, processed for immunofluorescence and imaged using phase contrast and fluorescence optics to observe melanosome and protein distribution (see Experimental Procedures). **a**) Representative images showing the distribution of melanosomes and proteins in cells. Scale bar = 20 $\mu$m. **b**) A bee swarm plot showing the extent of pigment dispersion (% total area) in cells shown in **a**. n = 20 cells for each condition. **** indicates significant difference p =< 0.0001 as determined by one-way ANOVA. n.s. indicates no significant difference. Bars between datasets indicate the populations that are subject of statistical testing. Bars withi datasets indicate the mean and 25$^{th}$ and 75$^{th}$ percentile of data. Data shown are from one experiment and the results are representative of 3 independent experiments. Source data for **b** are provided in the supplementary Source Data file.

## Supplementary Figure 10

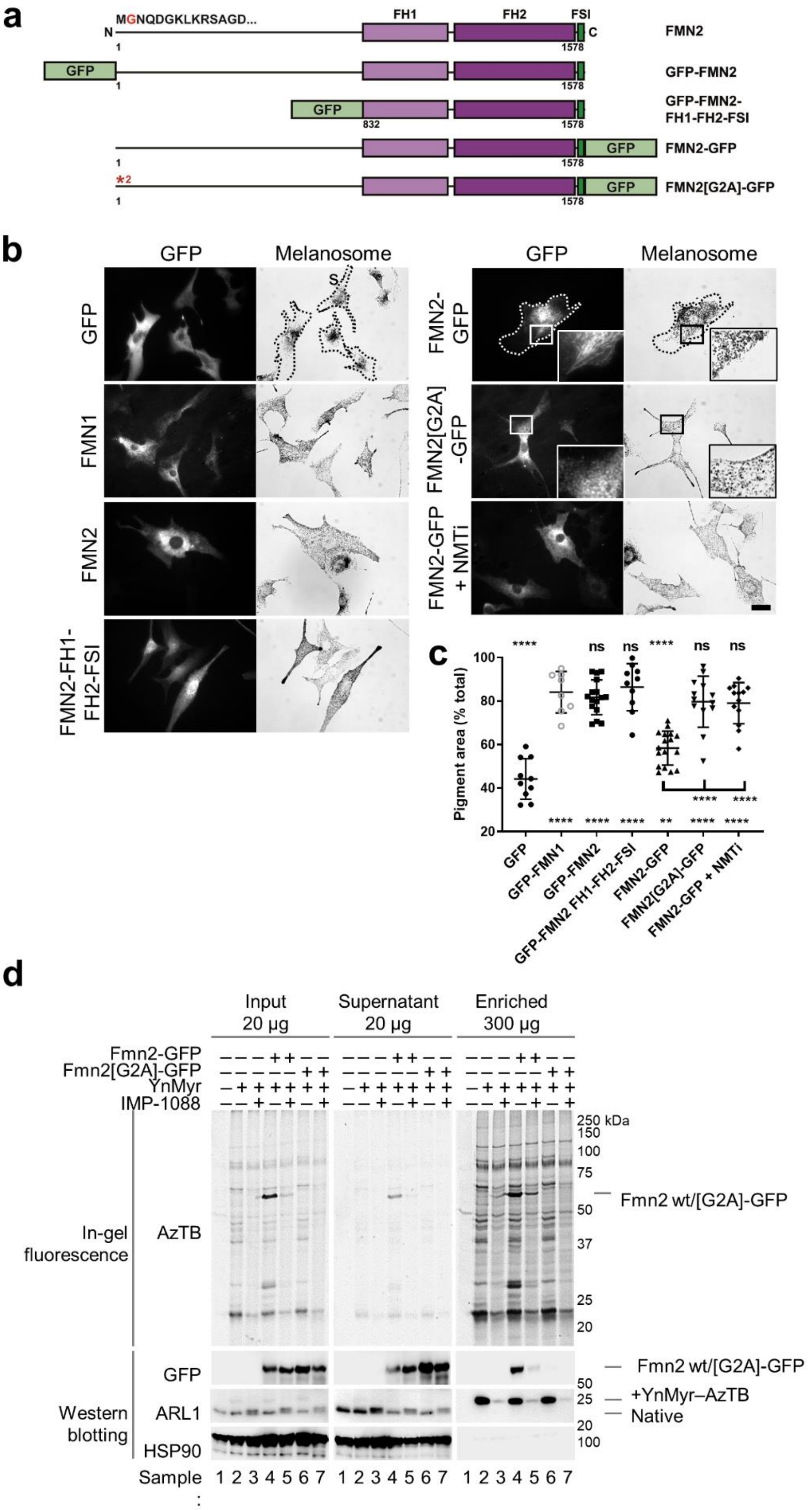


**Figure S10. Perturbation of N-myristoylation of FMN2 enhances its ability to rescue melanosome**

**transport in FMN1 deficient melanocytes.** melan-f cells were infected with adenoviruses expressing the indicated proteins in the presence/absence of NMTi IMP-1088. Cells were fixed and stained for immunofluorescence 7 hours later using anti-GFP antibodies, and the distribution of expressed protein (GFP) and melanosomes was recorded using a fluorescence microscope (see Experimental procedures). **a**) A schematic representation of the domain structure of murine FMN2 and the composition of truncations and chimeric proteins used in functional studies (**b**-**c**). Red text indicates the site of the glycine [2] that is predicted to undergo N-myristoylation. Numbers indicate amino acid boundaries. **b**) Representative images of cells expressing each protein. Dotted lines in FMN2-GFP images highlight the borders of cells. Scale bar represents 20 μm. **c**) A bee-swarm plot showing the extent of pigment dispersion (pigment area (% total)) in populations of cells in each condition. n (cells) = 10 (GFP), 8 (GFP-FMN1), 16 (GFP-FMN2), 10 (GFP-FMN2-FH1-FH2-FSI) 17 (FMN2-GFP), 13 (FMN2[G2A]-GFP) and 13 (FMN2-GFP + NMTi (IMP-1088 100nM)). **** and ** indicate significant difference p = < 0.0001 and p = < 0.01 as determined by one-way ANOVA. n.s. indicates no significant difference. Significance indicators above and below the dataset indicate differences between each condition and the positive (GFP-FMN1) and negative (GFP alone) controls. Horizontal bars linking dataset indicate other pairs of populations that show statistically significant differences. Results shown are representative of 3 independent experiments. Bars within datasets indicate the mean and 25$^{th}$ and 75$^{th}$ percentile of data. *FH*, formin homology; *FSI*, Formin-SPIRE interaction sequence. **d**) Purification of myristic acid analogue (YnMyr) labelled FMN2 proteins confirms N-myristoylation and the efficacy of inhibition strategies. HEK293a cells were transfected with plasmid vectors allowing expression of Fmn2-GFP and Fmn2[G2A]-GFP in the presence and absence of myristate analogue (YnMyr) and NMTi (IMP-1088). Cells were harvested 24 hours later and the myristoylated proteins purified (see Experimental procedures). Lysates (input), purified (enriched) and non-purified (supernatant) fractions were resolved by SDS-PAGE and YnMyr incorporation detected by in-gel fluorescence. Western blotting using antibodies specific for GFP shows expression of

Fmn2/Fmn2[G2A] and ARL1 and HSP90 antibodies confirm the efficacy and specificity of the labelling strategy. Source data for **c** and **d** are provided in the supplementary Source Data file.

## Supplementary Figure 11

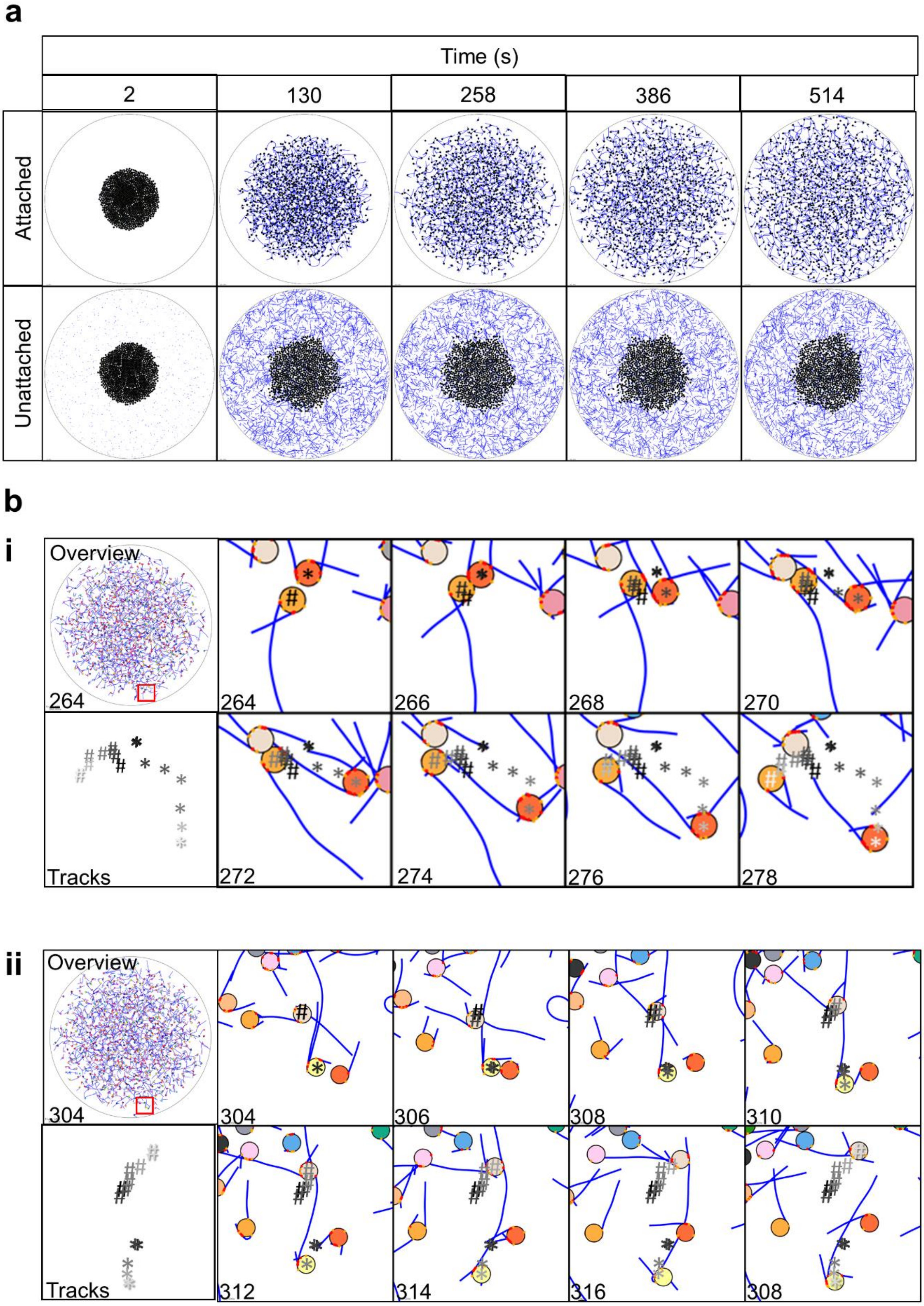


**Figure S11. Simulation showing the effect of AF polarity and anchoring on melanosome transport.**

Images collected from Cytosim simulations of melanosomes transport in model cells in with differing AF organisation (time from initiation of simulation is shown). Large black circles (diameter 46μm), small solid black circles (diameter 0.5μm) and blue lines, respectively represent the cell membrane, melanosomes and actin filaments. **a**) Attached and unattached image series show the results of transport simulations in which AF organisation is a) as described in Figure 9, i.e. with melanosomes attached via their -/pointed end to melanosomes with +/barbed ends grow outwards into the cytoplasm or b) with AFs unattached from melanosomes and free to diffuse throughout the cytoplasm. **b**) i and ii are enlarged areas (5 x 5μm) of cytoplasm (see red box in overview image for an indication of the position of the enlarged area within the cell area) from the 'attached' simulation showing examples of local dispersal of melanosomes at different time-points. In these images each melanosome has a different colour to aid tracking of individuals over time. Myosins, attached to melanosomes, are displayed as either red or orange depending on whether they are bound or unbound from actin filaments. Hash and asterisks indicate the centre of each melanosome over time with a gradient of colour from black to light grey indicating the changing position from early to late time-points. The complete tracks for each tracked melanosome are displayed in the tracks panel. Numbers in each panel indicate the elapsed time in seconds from the start point.

**References.**


1 Dettenhofer, M., Zhou, F. & Leder, P. Formin 1-isoform IV deficient cells exhibit defects in cell spreading and focal adhesion formation. *PLoS One* **3**, e2497, doi:10.1371/journal.pone.0002497 (2008).

2 Hume, A. N., Tarafder, A. K., Ramalho, J. S., Sviderskaya, E. V. & Seabra, M. C. A coiled-coil domain of melanophilin is essential for Myosin Va recruitment and melanosome transport in melanocytes. *Mol Biol Cell* **17**, 4720-4735, doi:10.1091/mbc.E06-05-0457 (2006).

3 Zhou, F., Leder, P. & Martin, S. S. Formin-1 protein associates with microtubules through a peptide domain encoded by exon-2. *Exp Cell Res* **312**, 1119-1126, doi:10.1016/j.yexcr.2005.12.035 (2006).

4 Nightingale, T. D., Pattni, K., Hume, A. N., Seabra, M. C. & Cutler, D. F. Rab27a and MyRIP regulate the amount and multimeric state of VWF released from endothelial cells. *Blood* **113**, 5010-5018, doi:blood-2008-09-181206 [pii]10.1182/blood-2008-09-181206 (2009).

5 Evans, R. D. *et al.* Myosin-Va and dynamic actin oppose microtubules to drive long-range organelle transport. *Curr Biol* **24**, 1743-1750, doi:10.1016/j.cub.2014.06.019 (2014).

6 Tarafder, A. K. *et al.* Rab27a targeting to melanosomes requires nucleotide exchange but not effector binding. *Traffic* **12**, 1056-1066, doi:10.1111/j.1600-0854.2011.01216.x (2011).

7 Hume, A. N. *et al.* Rab27a regulates the peripheral distribution of melanosomes in melanocytes. *J Cell Biol* **152**, 795-808 (2001).

8 Strom, M., Hume, A. N., Tarafder, A. K., Barkagianni, E. & Seabra, M. C. A family of Rab27-binding proteins. Melanophilin links Rab27a and myosin Va function in melanosome transport. *J Biol Chem* **277**, 25423-25430, doi:10.1074/jbc.M202574200 (2002).

9 Kukimoto-Niino, M. *et al.* Structural basis for the exclusive specificity of Slac2-a/melanophilin for the Rab27 GTPases. *Structure* **16**, 1478-1490, doi:10.1016/j.str.2008.07.014 (2008).

10 Ostermeier, C. & Brunger, A. T. Structural basis of Rab effector specificity: crystal structure of the small G protein Rab3A complexed with the effector domain of rabphilin-3A. *Cell* **96**, 363-374 (1999).

Uncropped blot from Figure 1d

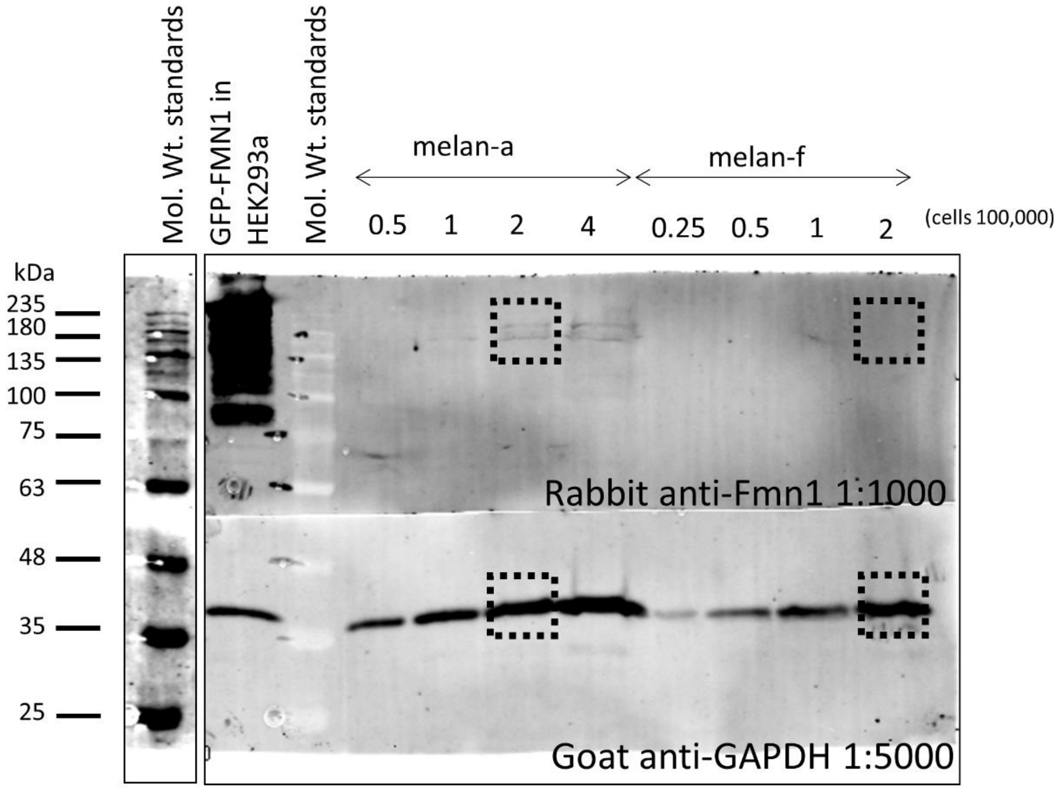


Boxes indicate the regions of the image that are included in Figure 1d

Uncropped blots from Figure 6

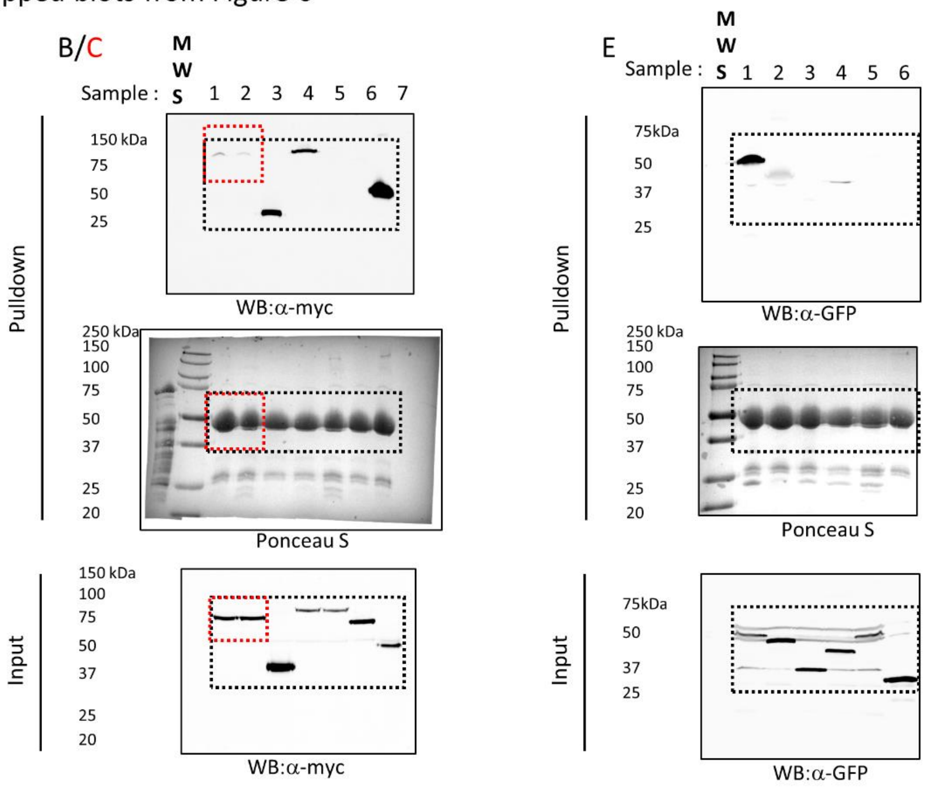


Full scans of blots and filters shown in Figure 6b, c and e. Black and red boxes indicate the areas shown in the final figure.

Uncropped blot from Supplementary Figure 4c (GAPDH)

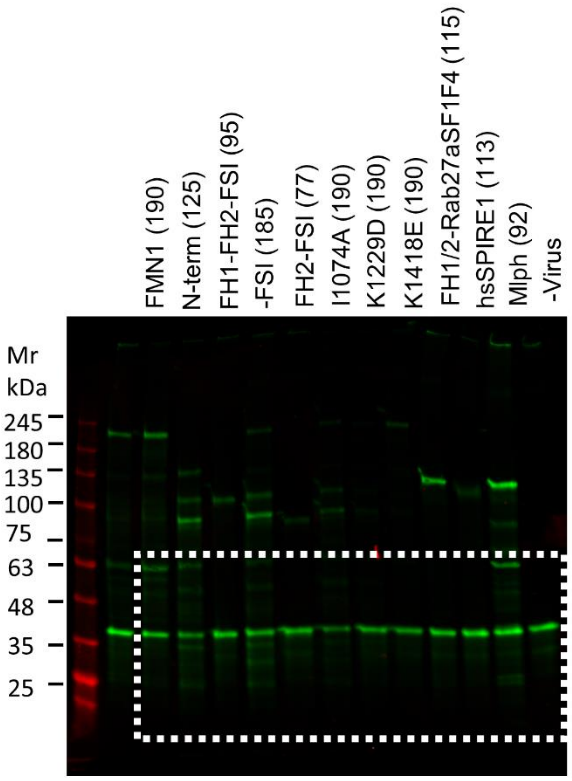


A composite image of the whole membrane showing the distribution of expressed GFP fusion proteins (bands of Mol. Wt. > 63kDa) and endogenous GAPDH (Mol. Wt. ~ 35kDa) in cell lysates (green signal) and the coomassie stained markers (red signal). The blot was probed sequentially with anti-GFP and then anti-GAPDH. The boxed area indicates the region of the image that is included as an inverted, contrast enhanced, grayscale image) in portion of Supplementary Figure 4c (GAPDH).

Uncropped blots from Supplementary Figure 5c

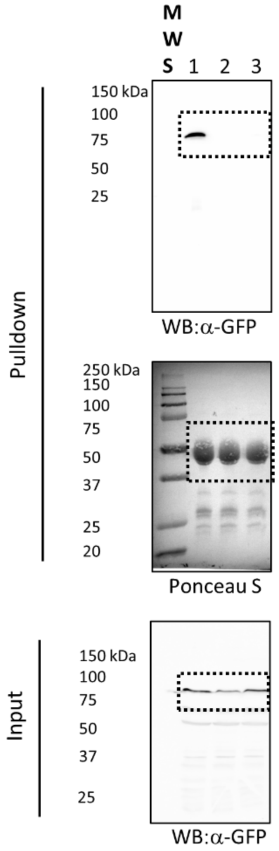


Full scans of blots and filters shown in Supplementary Figure 5c. Boxes indicate the areas shown in the final figure.

Uncropped blots from Supplementary Figure 6

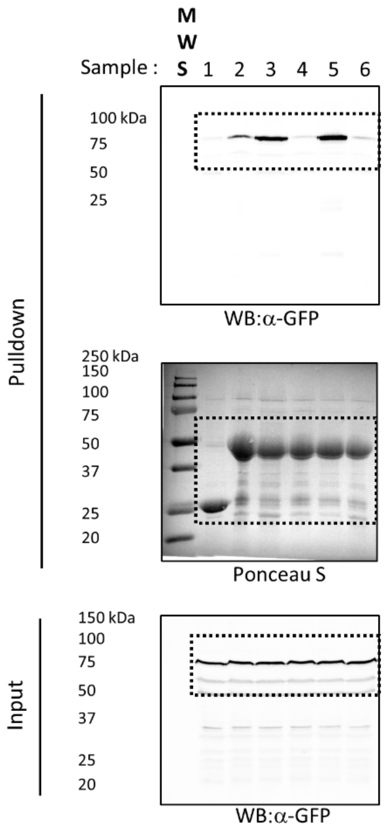


Full scans of blots and filters shown in Supplementary Figure 6. Boxes indicate the areas shown in the final figure.

Uncropped blot from Supplementary Figure 10d.

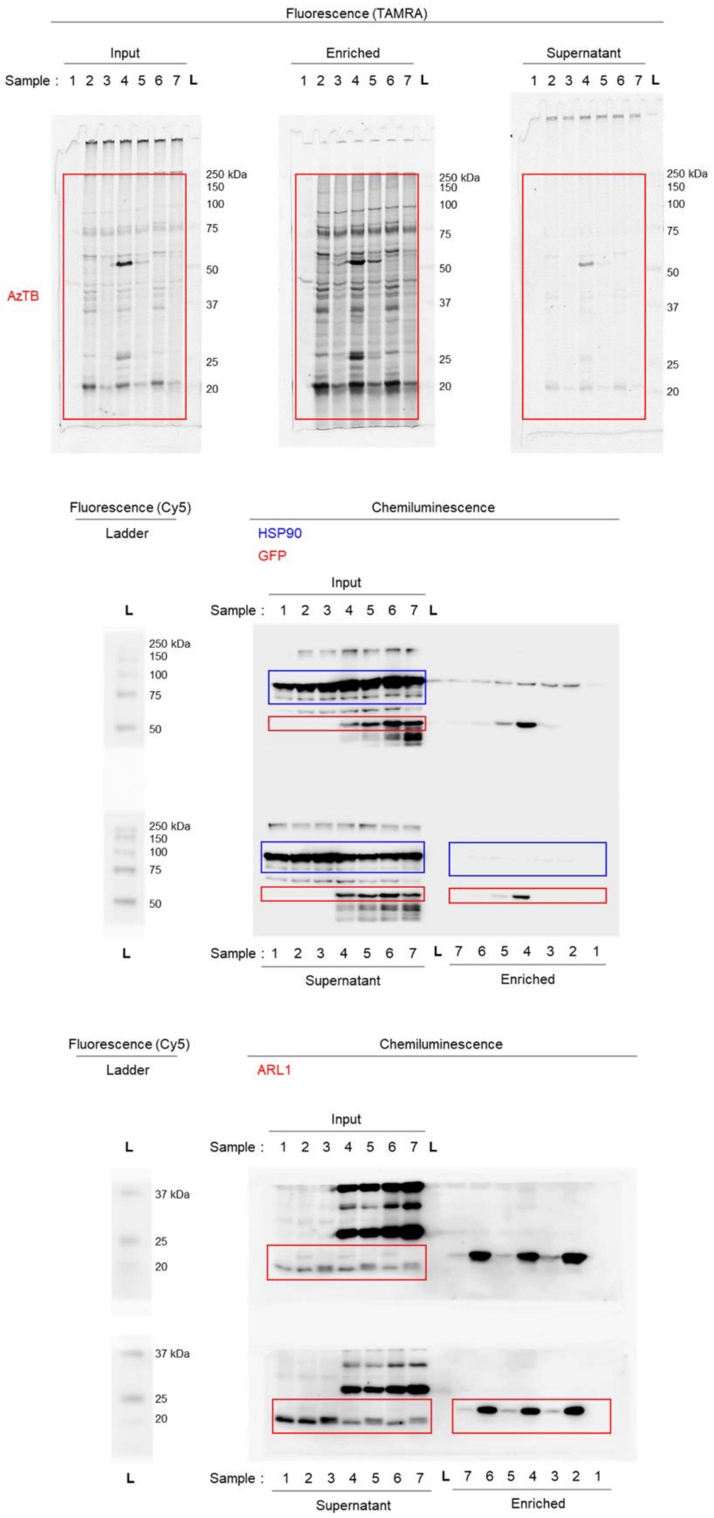


Red and blue boxes indicate the areas shown in final figures.
