# Supplementary material for: Rab27a co-ordinates actin-dependent transport by controlling organelle-associated motors and track assembly proteins
Source: Nat Commun. 2020 Jul 13;11:3495. doi: 10.1038/s41467-020-17212-6 (PMC7359353; doi:10.1038/s41467-020-17212-6)
Supplement: Supplementary file 3 — Description of Additional Supplementary Files [file 41467_2020_17212_MOESM3_ESM.pdf]

Description of Additional Supplementary Files

File Name: Supplementary Movie 1
Description: Supplementary Movie 1 is an image sequence showing the output of the 'attached' Cytosim simulation of melanosome transport in melanocytes see main text and Figure 9 for details. Companion to Figure S11A. Acquisition frame rate 0.5 sec-1. Playback frame rate 5 sec-1.

File Name: Supplementary Movie 2
Description: Supplementary Movie 2 is a high magnification detail from movie 1 showing the melanosome and actin filament movements in a small area of a model cell (melanosomes are shown individually coloured in order to aid tracking of individuals over time). This region corresponds to the red boxed region shown in the overview images in Figure S11B. Companion to Figure S11B. Acquisition frame rate 0.5 sec-1. Playback frame rate 5 sec-1.

File Name: Supplementary Movie 3
Description: Supplementary Movie 3 is an image sequence showing the output of the 'detached' Cytosim simulation of melanosome transport in melanocytes see main text and Figure 9 for details. C ompanion to Figure S11A. Acquisition frame rate 0.5 sec-1. Playback frame rate 5 sec-1.

File Name: Supplementary Code 1
Description: The melanosim model description document describes the model including a m odel overview, the properties and parameters used in modelling melanosomes, actin filame nts and the myosin-Va motors, the configuration code and references to literature.
