# Supplementary material for: Rab27a co-ordinates actin-dependent transport by controlling organelle-associated motors and track assembly proteins
Source: Nat Commun. 2020 Jul 13;11:3495. doi: 10.1038/s41467-020-17212-6 (PMC7359353; doi:10.1038/s41467-020-17212-6)
Supplement: Supplementary file 7 — Supplementary Code 1 [file 41467_2020_17212_MOESM7_ESM.docx]

Rab27A co-ordinates actin-dependent transport by controlling organelle-associated motors and track assembly proteins.

**Description of the Simulation**

03.2020

**Overview**

The simulation contains melanosomes, actin filaments, and myosin motors. The system is simulated in 2D, within a disc of diameter 50µm (“the cell”). In short, melanosomes are represented as spheres and the filaments as strings of points with bending elasticity and assembly/disassembly dynamics. The movements of melanosomes and the movements and deformations of filaments are calculated using overdamped Langevin dynamics as described previously ^1^. All events such as attachments and detachments of myosins are stochastic and modeled using a first order kinetics or a simple force-induced detachment (Bell’s law/Kramers theory). The different components of the simulation are described in this document in some details. For more information concerning the resolution of the equations using an implicit approach, please refer to our previous publication ^1^. For ultimate reference, please download our Open Source software (https://gitlab.com/f.nedelec/cytosim) and run it on your computer. The simulation configuration file copied here was used to simulate the wild-type case.

**Melanosomes**

A melanosome is characterized by a position and a constant radius R. Steric interactions between melanosomes penalize them for overlapping. It is implemented by adding a force between any two melanosomes that are less than 2R apart. This force is Hookean with a resting length 2R and a stiffness as specified in the parameter list (100 pN/µ
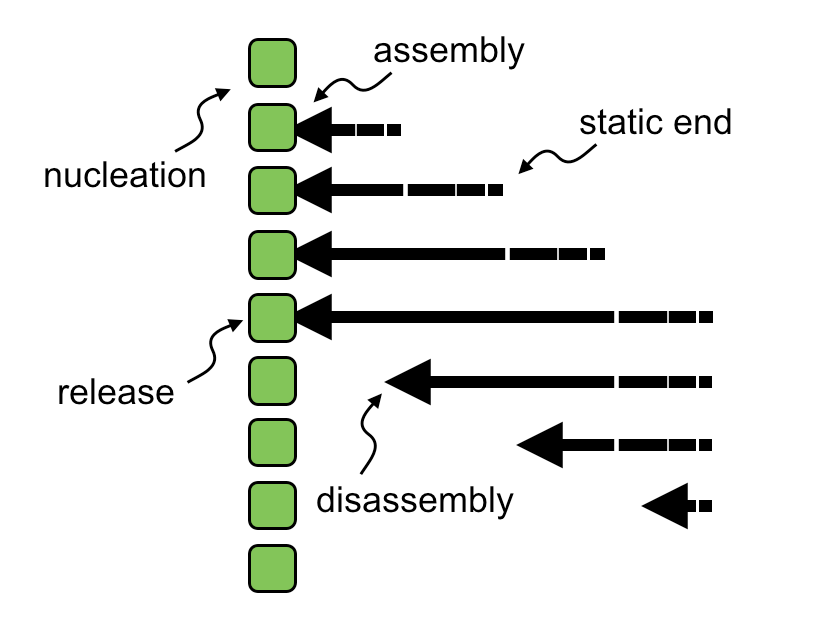
m). Each melanosome is associated with nucleating agents attached randomly on the surface of the melanosome. Any filament nucleated from the surface of the melanosome remains attached at its minus end, and is mechanically coupled by a Hookean link of zero resting length to the melanosome (200 pN/µm). In addition, myosin motors are also anchored to the melanosomes, also distributed randomly on the surface. Any filament captured by these motors is mechanically coupled by a Hookean link of zero resting length to the melanosome (200 pN/µm). The speed of the melanosome is proportional to the sum of the forces actin on it:
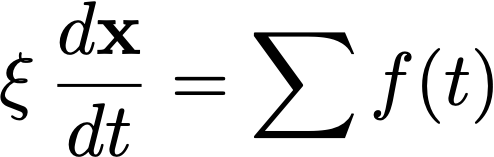


The force also includes a random component corresponding to Brownian motion as described (Nedelec and Foethke, 2007). The drag coefficient is obtained by Stokes’ law for an object of radius R embedded in a fluid of viscosity η:
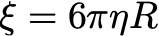


With the above equations, a melanosome can be pulled by forces generated by myosins on its surface, subject to steric interaction with other melanosomes.

Initially, all melanosomes are placed within a circle of diameter 16µm in the center of the cell.

**Actin**

Actin filaments are nucleated by agents called ‘nucleators’. Each nucleator can nucleate one filament at a time, and will stay attached to this filament until it completely depolymerize. The dynamics of the actin filaments is independent of force. The pointed/minus end is not dynamic. The barbed/plus end can undergo dynamic instability and can be found in two states: growing or shrinking. Upon nucleation, it will be growing. With a first order stochastic dynamics, it can transition to the shrinking state, and it will then disassemble at constant speed to eventually be removed when its length is below 10nm. There is no rescue. As the filament is removed the nucleator is able to nucleate a new filament.

Only unattached nucleators may nucleate new filaments. Thus, the number of filaments in the simulation is determined directly by the number nucleators, which is proportional to the number of melanosomes in the simulation. The fraction of filaments that are growing or shrinking are set simply by the castastrope rate and the ratio of growing and shrinking speed. Similarly, the length of filament follows an exponential distribution determined by these same three parameters. Typically, the number of shrinking filament is a small fraction of the total number of filaments, and the parameters were chosen to match the measured mean length of ~1µm.

Actin filaments are represented individually and mechanically behave like slender elastic beams. An interaction was included to confine the filaments to remain within the boundary of the cell. There is no limit on the number of myosin attached to a filament, and forces generated by them are additive.

**Myosin motors**

The Myosin-V motors are entities that may only bind one actin filament at any time. The motor is processive and directed toward the barbed/plus ends. It obeys a linear force-velocity relationship characterized by a unloaded speed and a stall force. The detachment increases with force, following Bells’ law / Kramers theory with a spontaneous rate and a characteristic force. The unloaded speed is from measurments ^2^, while the other parameters are expected values for molecular motors.

**Table of parameters**

Global

| Total time simulated | 1000 s | time-step is 2 ms. |
| --- | --- | --- |
| Geometry | Circular, diameter = 50 µm | Size of melanocyte |
| Viscosity | 0.1 N.s.m^-2^ | Measured viscosity range is 0.02 — 1 Pa.s |
| Temperature | kT = 0.0042 pN.µm | ~24°C |

Actin

| Bending elasticity | 0.1 pN.µm^2^ | Persistence length ~ 23 µm |
| --- | --- | --- |
| Confinement | Stiffness = 100 pN/µm | This is applied to points distributed every 100 nm, if they are outside the “cell”, confining all filaments to remain inside. |
| Initial length | 10 nm |  |
| assembly dynamics | Assembly = 0.1 µm/s  Disassembly = 0.5 µm/s  Castastrophe = 0.1 /s | After catastrophe, the end shrinks, and when the fiber eventually becomes shorter than 10 nm, it is deleted. |
| Pointed ends | no assembly/disassembly |  |
| Mobility | theoretical drag coefficient of a cylinder in infinite fluid | Caculated using the ‘hydrodynamic_radius’ with the formula page 2584, Table 1, last column, of Tirado and de la Torre. J. Chem. Phys 71(6) 1979 ^3^ |

Melanosomes

| Abundance | 1000 |  |
| --- | --- | --- |
| Nucleators  8 / melanosome | Nucleation rate = 0.1 / s  no detachment | A formin stays attached to the pointed end of the filament that it has nucleated, until it eventually vanishes. |
| Myosins  15 / melanosome | Binding rate = 10 / s  Binding range = 10 nm  Unbinding rate k_u_ = 0.3 / s  Unloaded speed v_0_= 0.3 µm/s  Stall force f_s_ = 3 pN  Unbinding force f_u_ = 3 pN | The velocity of the motors depends linearly on the force they experience:  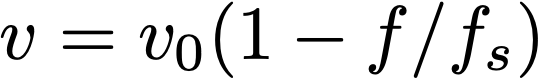  Unbinding is increased by force:  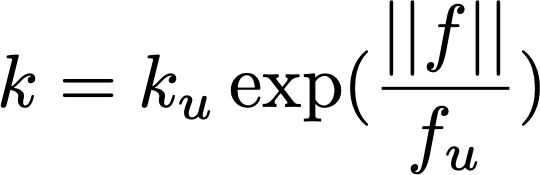 |
| Connection | Stiffness = 200 pN/nm | The actin filament nucleated by nucleators or captured by myosin are mechanically connected by a Hookean spring of null resting length. |
| Mobility | ξ ~ 0.19 pN s µm^-1^ | see equations for node motion in this document |
| Steric interaction | stiffness = 100 pN/nm | Hookean force with non-zero resting length |

Discretization parameters

| Time step | 2 ms | Results are independent of these values,  as long as they remain suitably small. |
| --- | --- | --- |
| Segmentation of fibers | 100 nm |  |

(in case of discrepancy, the configuration file below has the correct value)

Cytosim configuration file

(graphical parameters are omitted)

set simul system

{

viscosity = 0.1 % Pascal.second

time_step = 0.002 % second

steric = 1, 100 % enabled, with stiffness 200 pico-Newton/micrometer

}

set space cell

{

shape = circle

}

new cell

{

diameter = 50 % in micrometers

}

set solid melanosome

{

steric = 1

confine = inside, 100 % pico-Newton/micrometer

}

set fiber actin

{

rigidity = 0.1 % pico-Newton.micrometer^2

segmentation = 0.1 % micrometer: precision at which filaments are calculated

confine = inside, 100 % enable, stiffness in pico-Newton/micrometer

hydrodynamic_radius = 0.005 % micrometer; used to set drag coefficient of filaments

activity = classic % two-states model of dynamic instability

growing_speed = 0.10 % set by known average_length=growing_speed/catastrophe_rate

shrinking_speed = -0.5 % guess, but should be higher than 'growing_speed'

catastrophe_rate = 0.1 % guess, defines lifetime of filament

min_length = 0.010 % filaments shorter than this are deleted

persistent = 0 % delete filaments shorter than min_length

}

set hand myosin

{

binding_rate = 10 % 1/second; reasonable guess (measured value for kinesin)

binding_range = 0.01 % micrometer: distance of reach

unbinding_rate = 0.3 % 1/second; from Pierobon et al. BiophysJ 2009 (1/s)

unbinding_force = 3 % pico-Newton: reasonable guess (measured value for kinesin)

activity = move % uses a linear force-velocity relationship

unloaded_speed = 0.3 % micrometer/second; from Pierobon et al. BiophysJ 2009

stall_force = 3 % pico-Newton: reasonable guess (measured value for kinesin)

}

set hand nucleator

{

unbinding = 0, inf % never detach

activity = nucleate

% below '0.1' is the rate of nucleation.

% Filaments created at Length=0 with their barged (plus) end growing

nucleate = 0.1, actin, ( length=0; plus_end=grow; )

}

set single mel_nucleator

{

hand = nucleator

stiffness = 200 % pico-Newton/micrometer; estimated from force/molecular size

diffusion = 1 % micrometer^2/s; not used in wild-type situation

}

set single mel_myosin

{

hand = myosin

stiffness = 200 % pico-Newton/micrometer; estimated from force/molecular size

}

new 0 mel_nucleator

new 1000 melanosome

{

% below: 0.25 is the radius of melanophore (measured diameter of 0.5 micrometer).

% '6' is the number of nucleator per melanosome (measured range 2-10)

% ’15' is the number of myosin per melanosome (not measured)

sphere1 = center, 0.25, 6 mel_nucleator, 15 mel_myosin

position = disc 8 % initial position: within a circle of radius 8 micrometers

}

run system

{

nb_steps = 500000 % number of steps; total time is thus 1000 seconds

nb_frames = 1000

}

(end of configuration file)

References.

1 Nedelec, F. & Foethke, D. Collective Langevin dynamics of flexible cytoskeletal fibers. *New Journal of Physics* **9**, 427-450 (2007).

2 Pierobon, P. *et al.* Velocity, processivity, and individual steps of single myosin V molecules in live cells. *Biophys J* **96**, 4268-4275, doi:10.1016/j.bpj.2009.02.045 (2009).

3 Tirado, M. M. & J., G. d. l. T. Translational friction coefficients of rigid, symmetric top macromolecules. Application to circular cylinders. *Journal of Chemical Physics* **71** (1979).
