# Supplementary material for: Rab27a co-ordinates actin-dependent transport by controlling organelle-associated motors and track assembly proteins
Source: Nat Commun. 2020 Jul 13;11:3495. doi: 10.1038/s41467-020-17212-6 (PMC7359353; doi:10.1038/s41467-020-17212-6)
Supplement: Supplementary file 8 — Reporting Summary [file 41467_2020_17212_MOESM8_ESM.pdf]

nature research

Corresponding author(s): Alistair Hume

Last updated by author(s): 2020-05-29

Reporting Summary

Nature Research wishes to improve the reproducibility of the work that we publish. This form provides structure for consistency and transparency in reporting. For further information on Nature Research policies, see Authors & Referees and the Editorial Policy Checklist.

Statistics

For all statistical analyses, confirm that the following items are present in the figure legend, table legend, main text, or Methods section.

n/a	Confirmed	
☐	☒	The exact sample size (*n*) for each experimental group/condition, given as a discrete number and unit of measurement
☐	☒	A statement on whether measurements were taken from distinct samples or whether the same sample was measured repeatedly
☒	✓	The statistical test(s) used AND whether they are one- or two-sided<br>*Only common tests should be described solely by name; describe more complex techniques in the Methods section.*
☒	☐	A description of all covariates tested
☒	☐	A description of any assumptions or corrections, such as tests of normality and adjustment for multiple comparisons
☒	✓	A full description of the statistical parameters including central tendency (e.g. means) or other basic estimates (e.g. regression coefficient) AND variation (e.g. standard deviation) or associated estimates of uncertainty (e.g. confidence intervals)
☒	✓	For null hypothesis testing, the test statistic (e.g. *F*, *t*, *r*) with confidence intervals, effect sizes, degrees of freedom and *P* value noted<br>*Give P values as exact values whenever suitable.*
☒	☐	For Bayesian analysis, information on the choice of priors and Markov chain Monte Carlo settings
☒	☐	For hierarchical and complex designs, identification of the appropriate level for tests and full reporting of outcomes
☒	✓	Estimates of effect sizes (e.g. Cohen's *d*, Pearson's *r*), indicating how they were calculated

Our web collection on statistics for biologists contains articles on many of the points above.

Software and code

Policy information about availability of computer code

Data collection	Primer express v3.0.1, Step one v2.3,Qiagen Rotor-Gene Q Series 2.3.5, Axiovision 4.8, Leica LASX
Data analysis	SigmaPlot 12.3, ImageJ V2.0.0, Graphpad Prism 8,

For manuscripts utilizing custom algorithms or software that are central to the research but not yet described in published literature, software must be made available to editors/reviewers. We strongly encourage code deposition in a community repository (e.g. GitHub). See the Nature Research guidelines for submitting code & software for further information.

Data

Policy information about availability of data

All manuscripts must include a data availability statement. This statement should provide the following information, where applicable:

- Accession codes, unique identifiers, or web links for publicly available datasets
- A list of figures that have associated raw data
- A description of any restrictions on data availability

The data that support the findings of this study are available from the corresponding author upon reasonable request. The mass spectrometry proteomics data have been deposited to the ProteomeXchange Consortium via PRIDE partner repository with the dataset identifier PXD018083.

Field-specific reporting

Please select the one below that is the best fit for your research. If you are not sure, read the appropriate sections before making your selection.

☒ Life sciences ☐ Behavioural & social sciences ☐ Ecological, evolutionary & environmental sciences

Life sciences study design

All studies must disclose on these points even when the disclosure is negative.

Sample size
See figure legends in manuscript. Sample sizes were determined by the number of cells visible in images collected from samples.

Data exclusions
None

Replication
See figure legends in manuscript. All experiments were performed on at least 3 separate occasions and I confirm that all experiments gave the same successful results.

Randomization
Experiments were carried out on cultured cells therefore concerns over the allocation of participants to study groups is not relevant.
were controlled OR if this is not relevant to your study, explain why.

Blinding
Experimenters were not blinded to the indentity of samples or data during analysis. *and/or analysis. If blinding was not possible, describe why OR explain why blinding was not relevant to your study.*

Reporting for specific materials, systems and methods

We require information from authors about some types of materials, experimental systems and methods used in many studies. Here, indicate whether each material, system or method listed is relevant to your study. If you are not sure if a list item applies to your research, read the appropriate section before selecting a response.

Materials & experimental systems

n/a	Involved in the study
☐	☒ Antibodies
☐	☒ Eukaryotic cell lines
☒	☐ Palaeontology
☒	☑ Animals and other organisms
☒	☐ Human research participants
☒	☐ Clinical data

Methods

n/a	Involved in the study
☒	☐ ChIP-seq
☒	☐ Flow cytometry
☒	☐ MRI-based neuroimaging

Please see the tracked changes in the manuscript for details of animal procedures and ethical approvals.

Antibodies

Antibodies: Rabbit anti-FMN1 - Markus Dettenhofer (CEITEC - Central European Institute of Technology, Brno, Czech republic) described in doi:10.1371/journal.pone.0002497. Goat anti-GAPDH and goat anti-GFP - Sicgen Ab0049-200 and AB0020-200. Rabbit anti-GFP (Living Colors A.v. peptide antibody, polyclonal) - TakaraBio/Clontech 632376. Mouse anti-c-Myc 9E10, mouse anti-TRP1 antibody Ta99 and mouse-anti-human HSP90- Santa Cruz Biotechnology sc40, sc-58438 and sc-69703. Mouse anti-GFP - Roche 11814460001. Rabbit-anti-human ARL1 - Proteintech IRDye® 800CW Donkey anti-Rabbit IgG and IRDye® 800CW Donkey anti-Goat IgG Secondary Antibodies - LI-COR Biosciences 926-32213 and 926-32214. HRP donkey anti-rabbit IgG and HRP sheep anti-mouse IgG secondary antibodies - GE Healthcare Life Sciences 10794347 NA934 and 10094724 NXA931. Goat anti-mouse Alexa568 labelled secondary antibodies - Invitrogen A-11011. Cy5 conjugated donkey anti-mouse secondary antibodies - Dianova 715-175-151. HRP-conjugated anti-mouse and anti-rabbit secondary antibodies - Advansta R-05071-500 and R-05072-500.
Details of antibodies are including in revised tables S7 and S8.

Antibodies used
The conditions and details of antibodies used are described in the experimental procedures section of the manuscript.

Validation
Describe the validation of each primary antibody for the species and application, noting any validation statements on the manufacturer's website, relevant citations, antibody profiles in online databases, or data provided in the manuscript.

Antibody Validation (primary antibodies)
Rabbit anti-FMN1 See figure 7B in Dettenhofer 2008 PMID: 18560567
Goat anti-GAPDH See specificity statement from manufacturer website http://www.sicgen.pt/product/gapdh-polyclonal-antibody_1_5
Goat anti-GFP See specificity statement from manufacturer website http://www.sicgen.pt/product/gfp-polyclonal-antibody_1_1
Rabbit anti-GFP See specificity statement from manufacturer website https://www.takarabio.com/assets/documents/Certificate%20of%20Analysis/632377-PA923065.pdf
Mouse anti-c-Myc 9E10 See specificity statement from manufacturer website https://datasheets.scbt.com/sc-40.pdf
Mouse anti-TRP1 Ta99 See specificity statement from manufacturer website https://datasheets.scbt.com/sc-58438.pdf
Mouse monoclonal anti-GFP As shown in Figure S10d this antibody detects recombinant GFP protein only in HEK293a cells expressing GFP fusion proteins in western blotting. See also specificity statement from manufacturer website https://www.sigmaaldrich.com/content/dam/sigma-aldrich/docs/Roche/Bulletin/1/11814460001bul.pdf
Mouse-anti-human HSP90 See specificity statement from manufacturer website https://datasheets.scbt.com/sc-69703.pdf
Rabbit-anti-human ARL1 See specificity statement from manufacturer website https://www.ptglab.com/products/ARL1-Antibody-16012-1-AP.htm

Eukaryotic cell lines

Policy information about cell lines

Cell line source(s)
Sources of cell lines used are described in the experimental procedures section of the manuscript.

Authentication
Describe the authentication procedures for each cell line used OR declare that none of the cell lines used were authenticated.

Mycoplasma contamination
Confirm that all cell lines tested negative for mycoplasma contamination OR describe the results of the testing for mycoplasma contamination OR declare that the cell lines were not tested for mycoplasma contamination.

Commonly misidentified lines
(See ICLAC register)
Name any commonly misidentified cell lines used in the study and provide a rationale for their use.

Cell line sources - melan-f, melan-ash, melan-ln and melan-a melanocytes were from the Wellcome Trust Functional Genomics Cell Bank http://www.sgul.ac.uk/depts/anatomy/pages/WTFGCB.htm and HEK293a were from Thermofisher https://www.thermofisher.com/order/catalog/product/R70507#/R70507.

Authentication - Missing gene add-back/phenotype complementation experiments were carried out validate the identity of melan-f, melan-ash and melan-ln mutant cell lines e.g. expression of Rab27a but not other gene products restored dispersed melanosome distribution in Rab27a mutant melan-ash melanocytes. Conversely melan-a were authenticated as wild-type melanocytes by observation that they maintained dispersed cytoplasmic melanosome distribution that could be reversed by siRNA knockdown of Rab27a and other proteins involved in melanosome dispersion. HEK293a were authenticated in our laboratory on the basis of their ability to produce adenovirus when transfected with pAd adenovirus genome vectors.

Mycoplasma contamination - Cell lines were tested for rmycoplasm contamination at the time of derivation but not regularly thereafter.

Commonly misidentified lines - no commonly misidentified cell lines were used in this study
